# Mid1 is associated with androgen-dependent axonal vulnerability of motor neurons in spinal and bulbar muscular atrophy

**DOI:** 10.1038/s41419-022-05001-6

**Published:** 2022-07-13

**Authors:** Yosuke Ogura, Kentaro Sahashi, Tomoki Hirunagi, Madoka Iida, Takaki Miyata, Masahisa Katsuno

**Affiliations:** 1 Department of Neurology, Nagoya University Graduate School of Medicine, Nagoya, Japan; 2 Department of Anatomy and Cell Biology, Nagoya University Graduate School of Medicine, Nagoya, Japan; 3 Department of Clinical Research Education, Nagoya University Graduate School of Medicine, Nagoya, Japan

**Keywords:** Neurodegeneration, Motor neuron disease

## Abstract

Spinal and bulbar muscular atrophy (SBMA) is an adult-onset hereditary neurodegenerative disease caused by the expansions of CAG repeats in the *androgen receptor* (*AR*) gene. Androgen-dependent nuclear accumulation of pathogenic AR protein causes degeneration of lower motor neurons, leading to progressive muscle weakness and atrophy. While the successful induction of SBMA-like pathology has been achieved in mouse models, mechanisms underlying motor neuron vulnerability remain unclear. In the present study, we performed a transcriptome-based screening for genes expressed exclusively in motor neurons and dysregulated in the spinal cord of SBMA mice. We found upregulation of *Mid1* encoding a microtubule-associated RNA binding protein which facilitates the translation of CAG-expanded mRNAs. Based on the finding that lower motor neurons begin expressing Mid1 during embryonic stages, we developed an organotypic slice culture system of the spinal cord obtained from SBMA mouse fetuses to study the pathogenic role of Mid1 in SBMA motor neurons. Impairment of axonal regeneration arose in the spinal cord culture in SBMA mice in an androgen-dependent manner, but not in mice with non-CAG-expanded *AR*, and was either exacerbated or ameliorated by *Mid1* overexpression or knockdown, respectively. Hence, an early Mid1 expression confers vulnerability to motor neurons, at least by inducing axonogenesis defects, in SBMA.

## Introduction

Spinal and bulbar muscular atrophy (SBMA) or Kennedy’s disease is an adult-onset X-linked hereditary neurodegenerative disease. SBMA is accompanied by the degeneration of lower motor neurons in the spinal cord and specific brainstem motor nuclei and atrophy of skeletal muscle resulting in progressive motor impairment [1]. Patients with SBMA carry trinucleotide CAG-repeat expansion which encodes the polyglutamine (polyQ) tract in the first exon of the *androgen receptor* (*AR*) gene [2].

Androgen-dependent nuclear accumulation of mutant AR protein causes neurological symptoms [3], typically affecting males. The nuclear accumulation of pathogenic AR has been shown to induce transcriptional dysregulation and subsequent defects in numerous cellular functions resulting in cell death of motor neurons [4–10]. Motor neurons in the mouse model of SBMA have been shown to exhibit axonal dysfunction and atrophy without noticeable cell death [3, 7], suggesting that axonal dysfunctions can induce symptoms. The early, presymptomatic dysregulation of axonal transport by pathogenic AR has been proposed as a causative factor for axonal defects [9, 11, 12]. Hence, further exploration of the mechanisms of early axonal vulnerability is essential for elucidating SBMA pathogenesis.

Androgen-dependent regulation of axonogenesis in motor neurons has been extensively studied so far [13]. Androgen has been shown to promote axonogenesis by upregulating brain-derived neurotrophic factor (BDNF) in motor neurons [14] and inducing the expression of *cpg15/neuritin* [15]. Inversely, pathogenic AR impairs axonal transport and the BDNF-dependent growth of neurites in cell culture [11, 12].

Several mechanisms have been proposed for how pathogenic AR disrupts axonal transport. The aggregates of pathogenic nuclear AR cause toxicity, which downregulates the gene expression of *dynactin1* and impairs retrograde axonal transport driven by dynein [9]. Non-aggregated and oligomeric pathogenic AR induces phosphorylation-mediated inhibition of kinesin via c-Jun N-terminal kinase activation, leading to impairment of anterograde axonal transport [12]. Hence, the previous studies suggest that transcriptional dysregulation and post-transcriptional regulation by pathogenic AR might be involved in the disruption of axonal transport and axonogenesis in SBMA. Translation of dysregulated cytoplasmic mRNA has been increasingly considered to be crucial for the pathogenesis of neurodegenerative diseases, including polyQ diseases [16]. Pathogenic proteins with a polyQ expansion have been shown to induce endoplasmic reticulum stress, which inhibits eukaryotic translation initiation factor 2 (eIF2), leading to cell death of vulnerable neurons in cell culture [17, 18]. Midline 1 (human MID1 and its mouse orthologue Mid1), an ubiquitin E3 ligase, was shown to form a microtubule-associated ribonucleotide complex with purine-rich sequence motif in mRNAs [19], which promotes translation of mRNAs harboring a CAG-repeat via mechanistic target of rapamycin (mTOR) signaling [20, 21]. These CAG-repeat mRNAs include AR with a non-expanded CAG repeat [22]. However, the cellular processes through which pathogenic AR causes translational dysregulation in SBMA remains elusive.

Here, we performed a transcriptome-based screening of genes and identified *Mid1*, a development-related gene, specifically expressed in the spinal motor neurons and upregulated in the spinal cord of SBMA mice. Using spinal cord slice cultures from the embryos of SBMA mice, we found that overexpression of *Mid1* increased the pathogenic AR protein levels, thereby inducing the androgen-dependent impairment of axonogenesis. Our findings indicate a mechanism for the *Mid1*-associated axonal vulnerability in SBMA motor neurons.

## Materials and methods

### DNA constructs

The plasmids pCR3.1-*AR-24Q* and pCR3.1-*AR-97Q*, which drive expression of full-length human AR containing 24 and 97-expanded CAGs, respectively, under the control of a cytomegalovirus enhancer and a chicken *β-actin* promoter as previously described [23] were used in the current study. Mouse *Mid1* cDNA was amplified from the cDNA obtained from mouse spinal cord on embryonic day 14.5 (E14.5) and was cloned into the *Not*I/*Eco*RI site of pcDNA3.1-*mycHisC* to create pcDNA3.1-*Mid1-mycHisC*. Infusion system (Invitrogen) was used for cloning *Mid1* cDNA together with the sequence encoding the self-cleaving peptide 2 A into pLenti-*GFP(C)*, resulting in pLenti-*GFP-2A-Mid1*. The plasmids for shRNA expression were obtained by inserting the target sequences into the *Age*I/*Eco*RI site of pLKO.1 puro (Addgene). The target sequences of shRNA for mouse *Mid1* and human *AR* were GACTTGCGTTACTTGTGAA [24] and GAAAGCACTGCTACTCTTCAGCATTATTCCA [25], respectively.

### Cell culture and transfection

Mouse NSC34 motor neuron-like cells (kindly provided by N.R. Cashman, University of British Columbia, Vancouver, Canada) were grown in Dulbecco’s Modified Eagle’s Medium (DMEM) supplemented with 10% fetal bovine serum (FBS). In addition, cells were maintained in a 37 °C incubator with a humidified atmosphere of 95% air/5% CO_2_. The plasmids were transfected using OPTI-MEM (Gibco) and Lipofectamine 2000 (Invitrogen). Ten hours after lipofection, the culture medium was changed to DMEM containing 2% FBS and 50 nM dihydrotestosterone (DHT). In the case of cycloheximide (CHX) treatment experiments, the culture medium was changed to DMEM containing 2% FBS and 80 μg/mL CHX, 24 h after lipofection.

### Immunoblotting

Protein isolation was performed using Cellytics MT Cell Lysis Reagent (Sigma-Aldrich). We separated equal amounts of protein on 5–20% SDS-PAGE gels (FUJIFILM Corp.) and transferred them to Hybond-P membranes (GE Healthcare). The following primary antibodies were used with indicated dilutions: rabbit anti-AR pAb (H-280, Santa Cruz Biotechnology., 1:2000), mouse anti-Gapdh mAb (Millipore, 1:5000), rabbit anti-MID1 pAb (Abcam, 1:1000), rabbit anti-PP2Ac pAb (Cell Signaling Technology, 1:1000), rabbit anti-GFP pAb (MBL, 1:1000). The density of each band was quantified using ImageJ software (NIH, Bethesda, MD, USA). For RNA pull-down assay, cell lysate containing ~300 μg of total protein derived from HEK293T cells overexpressed with Mid1 was incubated for 1 h with 3 μg of in vitro-transcribed RNA biotinylated with Biotin-16-UTP (Sigma-Aldrich). It was followed by incubation with Dynabeads M-280 streptavidin (Invitrogen) for 1 h at 4 °C and washed with wash buffer three times.

### Transcriptome data

Previously published microarray data on Gene Expression Omnibus, GSE39865 [4], was normalized using Robust Multichip Analysis of Bioconductor based on R (https://www.r-project.org). The resultant data was subjected to the differential gene expression analysis using an integrated web application for Differential Expression and Pathway analysis (iDEP) [26]. Cell type specificity of candidate genes was examined online using single-cell transcriptional atlas [27] (http://spinalcordatlas.org).

### RT-PCR

Total RNA was extracted from NSC34 cells or spinal cord slice culture using TRIzol (Invitrogen) and RNeasy Mini Kit (QIAGEN). The extracted RNA was then reverse-transcribed into first-strand cDNA using ImProm-II Reverse Transcription system (Promega). Quantitative PCR was performed using THUNDERBIRD SYBR qPCR Mix (TOYOBO), and the amplified products were detected with the iCycler system (Bio-Rad Laboratories). The following primers were used for PCR: *Mid1* forward/reverse, 5'-*AGCCCTTTACAGGCCATCG*-3'/5'-*AGATTAACTGGTCATCGGTCACA*-3'*; AR* forward/reverse, 5'-*CGGAAGCTGAAGAAACTTGG*-3'/5'-*ATGGCTTCCAGGACATTCAG*-3*'; β*_*2*_*-microglobulin* forward/reverse, 5'-*CTGACCGGCCTGTATGCTAT*-3'/5'-*CCGTTCTTCAGCATTTGGAT*-3'. The gene expression of *beta2-microglobulin* was used for normalization.

### RNA immunoprecipitation

RNA immunoprecipitation (RIP) was performed using the total cell lysates from HEK293T cells co-expressing His-tagged *Mid1* and *AR-24Q* or *AR-97Q* and anti-His-tag mAb-Magnetic beads (MBL) (or nonspecific Mouse IgG2a (isotype control)-Magnetic beads (MBL) antibody) for 1 h at 4 °C and washed with Cellytics MT for three times, followed by RNA purification using TRIzol (Invitrogen) and RNeasy Mini Kit (QIAGEN) and examined by RT-PCR using rTaq DNA polymerase (TaKaRa) and the *AR* forward/reverse primers mentioned above.

### Immunofluorescence

Cultured spinal cord slices were fixed with 4% PFA, immersed in 20% sucrose, embedded in OCT compound, and cryo-sectioned (16 μm thickness). Sections were incubated overnight at 4 °C with the following primary antibodies with the indicated dilution: rabbit anti-MID1 pAb (ab70770, Abcam, 1:400); mouse anti-Islet1&2 mAb (39.4D5, DSHB, 1:100); rabbit anti-AR (D6F11, Cell Signaling Technology, 1:400) mAb; mouse anti-non-phosphorylated NF-H mAb (SMI32, BioLegend, 1:2000); rabbit anti-Pnpt1 (Invitrogen, 1:200). After PBS washes, the sections were incubated for 1 h at room temperature with the following secondary antibodies: Donkey anti-Rabbit IgG-Alexa488 and Donkey anti-Mouse IgG-Alexa546 (Invitrogen, 1:1000). After incubation, the sections were counterstained with DAPI and mounted with a mounting solution. Images were acquired using CoolSNAP CCD camera (Photometrics) assembled on BX60 upright microscope (Olympus).

### Virus production

Lentiviral particles were produced in HEK293T cells by transfection of pLenti-*GFP(C)*, pLenti-*GFP-2A-Mid1*, pLKO.1-*contol shRNA* (SHC016, Sigma), pLKO.1-*Mid1 shRNA,* or pLKO.1-*human AR shRNA* together with pLP1, pLP2, and pVSV-G (Invitrogen) using Lipofectamine 2000 (Invitrogen). The lentiviral-containing supernatants were collected 48 h after transfection and were concentrated by ultracentrifugation using Lenti-X Concentrator (Takara Bio USA). The viral titers were measured using Lenti-X qRT-PCR Titration Kit (Applied Biological Materials). The final concentration of lentiviral particles in the culture medium was ~5.0 × 10^7^ IU/mL.

### Maintenance of transgenic animals

AR-97Q transgenic mice expressing full-length AR containing 97-expanded CAGs under the control of a cytomegalovirus enhancer and a chicken *β-actin* promoter were generated in our previous study [3]. The mice were bred, maintained on a C57BL/6 J mouse background in the animal facility and were genotyped by PCR amplification using DNA extracted from the tail. The following primers were used for PCR: *AR* forward/reverse, 5'-*CTTCTGGCGTGTGACCGGCG*-3'/ 5'-*TGAGCTTGGCTGAATCTTCC*-3'; *Sry* forward/reverse, 5'-*TGGGACTGGTGACAATTGTC*-3'/5'-*GAGTACAGGTGTGCAGCTCT*-3'. Mice were fed with a standard diet and maintained on a 12 h light/dark cycle. Pregnant mice were obtained by crossing female AR-97Q transgenic mice with wild-type C57BL/6 J male mice.

### Spinal cord slice culture

Cross-sectional slices of the spinal cord (250–300 μm thickness) were prepared as previously described for embryonic cerebral wall [28, 29] and cerebellar primordium [30]. As a prerequisite for the experiment, silicon rubber was solidified (5–10 mm thick and transparent) in a Petri dish by mixing KE-103 and CAT-103 (Shin-Etsu Chemical). Embryos were dissected in a Petri dish containing DMEM/F12, and vertebral bones and meninges were removed by tweezers. The naked spinal cords were cooled on ice for 2–3 h during the genotyping, transferred to the previously solidified silicon rubber, and sliced manually using micro-knives purchased from Alcon (V-Lance Ophthalamic corneal/scleral knife, 20 Gage). Using a 200 μL Pipetman (Gibson) with a large orifice (1–2 mm) tip, slices were transferred with ~200 μL enriched medium [DMEM/F12 supplemented with 5% horse serum, 5% FBS, N2 (1:100, Thermo Fisher Scientific)] to 35 mm dish (IWAKI). Slices were mixed with ~200 μL of 2 × type I collagen solution (1.5–1.6 mg/ml in distilled water, 5 × DMEM/F12 and neutralizing buffer; Cell matrix IA; Nitta Gelatin), prepared according to the manufacturer’s instructions, and was kept on ice until use. The gel (final concentration 0.7–0.8 mg/ml) was immediately spread on the dish base using a pipette tip. The transverse plane of the spinal cord slices was arranged horizontally to the dish base using a tungsten needle. The dishes were placed in an incubator (37 °C; 5% CO_2_, 40% O_2_) to allow for further gel solidification (5–10 min). An enriched medium (1 ml) was added to the gel and spread over the entire dish surface. Lentivirus (5.0 × 10^7^ IU/mL in a final concentration) or DHT (50 nM in a final concentration) was directly added to the dish at the start of culture (day 0). Images of cultured spinal cord slices were acquired using ORCA-flash 4.0 v3 camera (Hamamatsu Photonics) assembled on an IX71 inverted microscope (Olympus).

### Statistical analysis

We analyzed the data with unpaired two-sided *t*-test to compare two groups and analysis of variance (ANOVA) with Tukey’s test for multiple comparisons using Prism 8 Software (GraphPad). We considered *p* < 0.05 to indicate statistical significance. The data were checked for normality using Shapiro-Wilk test and for equality of variances using *F*-test. In the microarray data analysis, the false discovery rate (FDR) < 0.05 was considered to be statistically significant.

## Results

### *Mid1* stands as a candidate gene for motor neuron vulnerability in SBMA

**Fig. 1:**
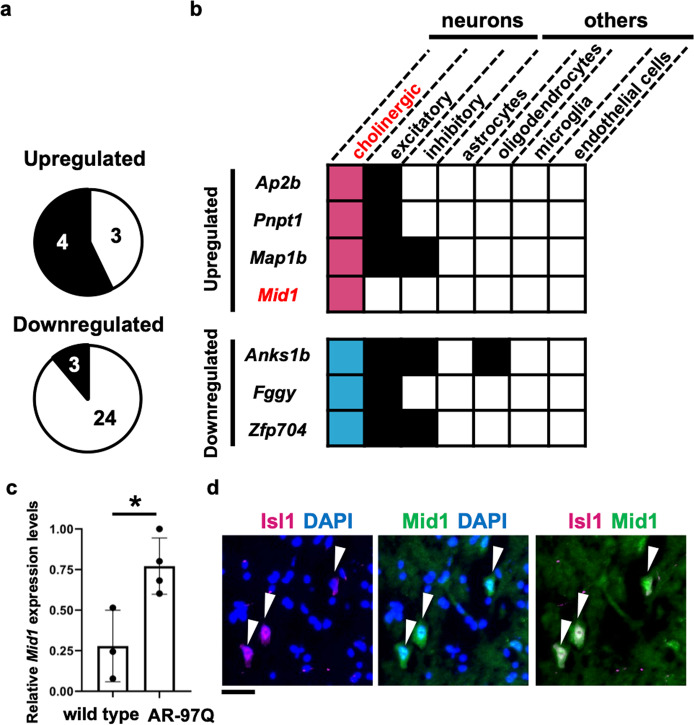
*Mid1* as a candidate gene associated with motor neuron vulnerability in SBMA. **a** The spinal cord transcriptome data of SBMA mice [4] contained seven upregulated and 27 downregulated genes. Analyses of these genes in single-cell transcriptional atlas [27] identified four upregulated and three downregulated genes expressed in the cholinergic neurons in the spinal cord of adult mice. **b** A binary matrix summarizing the expression of the genes in the other cell types in the spinal cord using the single-cell transcriptional atlas [27]. The expression was indicated by the filled matrices. **c** Relative quantification of *Mid1* expression in the spinal cord of AR-97Q mice at 12 weeks of age (*p < 0.05, unpaired two-tailed two-sample *t*-test). **d** Immunostaining of Mid1 in the spinal cord of the mouse at 10 weeks of age. Isl1 is a motor neuron marker. Scale bar 50 μm.

To identify genes involved in the degeneration of motor neurons, we reanalyzed the transcriptomes from the spinal cord of AR-97Q mice at different ages: 7–9, 10–12, and 13–15 weeks [4] and combined them with the single-cell transcriptional atlas [27] to extract genes specifically dysregulated in the spinal motor neurons of AR-97Q mice. Among the genes whose expression was significantly altered by more than 2-fold in AR-97Q mice compared to the control AR-24Q mice, we identified four upregulated genes (two genes during 7–9 weeks and two genes during 10–12 weeks) and three downregulated genes (all genes during 7–9 weeks) expressed in cholinergic neurons which express choline acetyltransferase, a motor neuron marker (Fig. 1a; Supplementary Table 1).

Selected vulnerability of lower motor neurons within the central nervous system in SBMA suggests that genes exclusively expressed in motor neurons are involved in the pathogenesis of SBMA. Therefore, we examined the expression of the candidate genes in the other cell types in the spinal cord using the single-cell transcriptional atlas [27] to determine their properties for motor neuron specificity. Among the differentially expressed genes we identified, *Mid1*, which encodes a microtubule-associated RNA binding protein [19], had the largest fold change (Supplementary Table 1). In addition, the expression of *Mid1* was restricted to the spinal motor neurons according to the single-cell transcriptional atlas (Fig. 1b). We used quantitative RT-PCR to confirm the upregulation of *Mid1* in the spinal cord at the early symptomatic stages of AR-97Q mice (Fig. 1c). Furthermore, immunostaining confirmed that Mid1 expression is specific to the spinal motor neurons in AR-97Q mice (Fig. 1d), suggesting that the upregulation of *Mid1* associated with pathogenic AR selectively occurs in the motor neurons. Moreover, both spinal cord samples from SBMA patients and previously published RNA-seq data using induced pluripotent stem cell (iPSC)-derived motor neurons from SBMA patients [31] displayed an increased level of Mid1 (Supplementary Fig. 1), confirming the similar trends in human. We also examined the expression of *Pnpt1*, which had the second-largest fold change, using immunofluorescence, but the expression pattern of it was not motor neuron-specific in the spinal cord (Supplementary Fig. 2). Therefore, we focused on *Mid1* henceforth.

### Mid1 increases pathogenic AR protein in a cellular model of SBMA

**Fig. 2:**
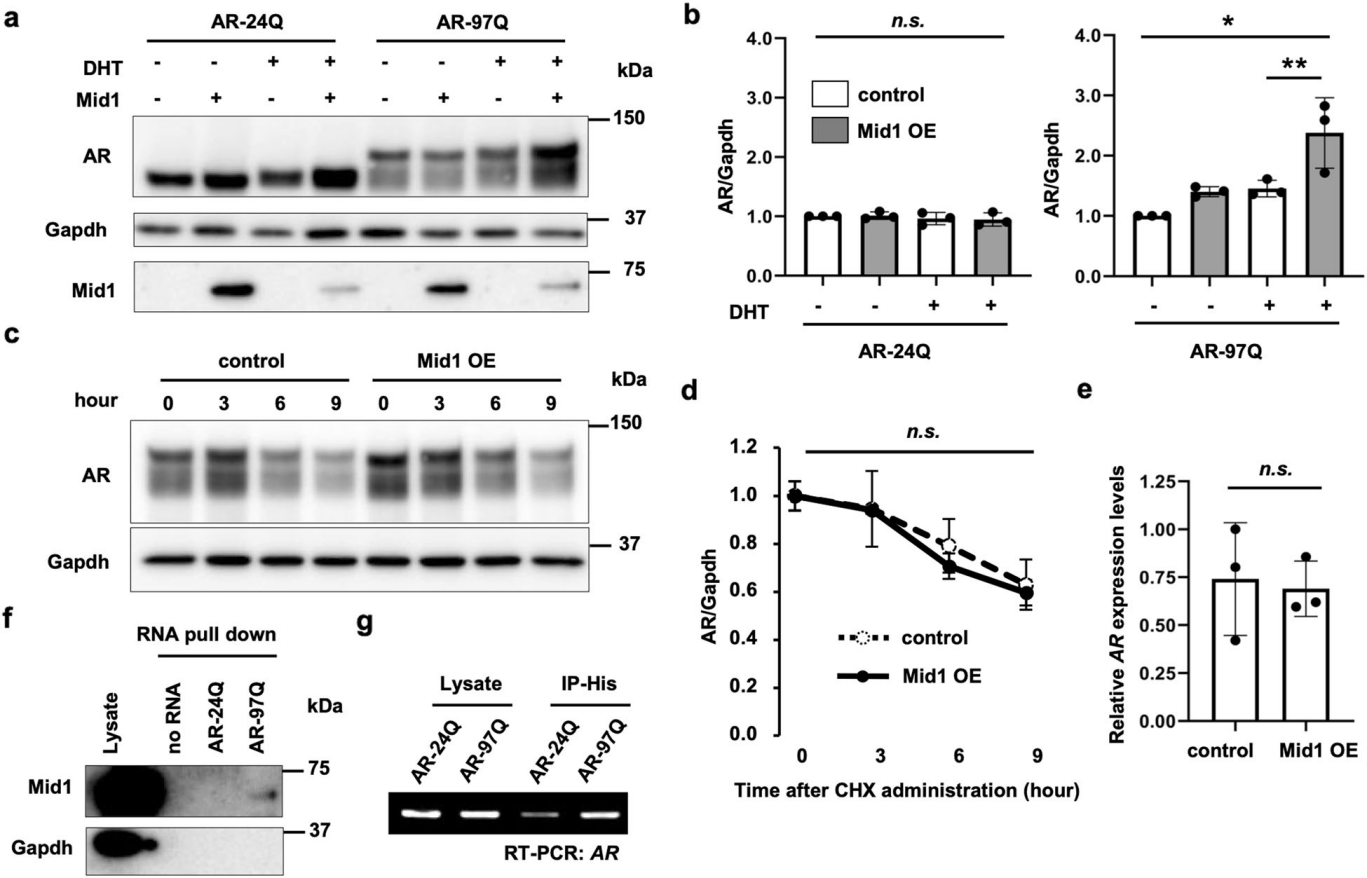
Mid1-induction of pathogenic AR protein in an SBMA cellular model. **a** Immunoblots showing levels of human AR protein in NSC34 cells expressing AR-24Q or AR-97Q, which were cultured with or without DHT treatment and transfected with *Mid1*-expressing or empty vector. **b** Quantitative densitometry analyses of human AR levels in **a** (*n* = 3 samples per group). The data were normalized to the AR levels without DHT treatment and *Mid1* transfection. Significant difference was detected by Tukey’s multiple comparisons test (*p < 0.005, **p < 0.05) after one-way ANOVA (*p < 0.005 for AR-97Q samples). **c** Immunoblots showing the time course of human AR protein levels in NSC34 cells expressing AR-97Q and transfected either with *Mid1* or empty vector. Hours after the administration of CHX are shown. **d** Quantitative densitometry analyses of the time course of human AR protein levels in **e** (*n* = 3 samples per group). The data were normalized to the AR/Gapdh level at 0 h. No significant difference was detected by Two-way ANOVA. **E** Quantification of human AR expression levels in NSC34 cells transfected with *Mid1*-expressing vector or empty vector (*n* = 3 samples per group; Wilcoxon rank-sum test). n.s. not significant. **f** Immunoblots showing Mid1 in the total cell lysate (loading control), RNA-protein pull-down of Mid1 with no RNA, RNA containing 24 or 97 CAG repeats (AR-24Q, AR-97Q). Gapdh was used as a negative control. **g** An agarose gel showing RT-PCR products of *AR* from total cell lysate (loading control) and RIP products of HEK293T cells overexpressing *AR-24Q/AR-97Q* and *Mid1*.

Previous studies on cell lines have shown that Mid1 binds to several mRNAs containing a CAG-repeat in a length-dependent manner [20, 21] to increase their translation. Although Mid1 protein is reported to bind to the CAG repeat of *AR* mRNA to regulate its translation [22], whether the translation of pathogenic AR with an expanded CAG repeat can similarly be increased was not known. Therefore, we tested the effects of overexpressing Mid1 on the level of pathogenic AR protein in the mouse Neuroblastoma-Spinal Cord hybrid (NSC) 34 cell line [32] (Fig. 2a, b). As we performed a short-term culture within 24 h, administration of DHT resulted in only moderate increases in AR protein levels. The protein levels of Mid1 was decreased by treatment with DHT (Fig. 2a), possibly due to a negative regulation of Mid1 by activated AR regardless of expanded polyglutamine repeats [22]. In this condition, overexpression of Mid1 increased the protein level of AR-97Q, but not AR-24Q, in the presence of DHT. Hence, the results suggested that the increase of AR protein levels by Mid1 overexpression depended on both the length of a CAG repeat and DHT levels.

The increase of AR protein levels could result from an increase in translation, a decrease in protein degradation, and/or an increase in transcription. CHX pulse-chase experiment ruled out the second possibility: Mid1 overexpression did not change the time course of pathogenic AR protein levels under CHX treatment (Fig. 2c, d), suggesting that the degradation of pathogenic AR protein was not affected by Mid1. To exclude the third possibility, the transcript level of *AR* transgene, which was driven by constitutive CAG promoter [3], was measured with quantitative RT-PCR. The result showed no change in *AR* transcript levels after Mid1 overexpression, confirming that AR was not upregulated at the transcript level (Fig. 2e). Therefore, an increase in translation appears to be a plausible mechanism for the rise of AR protein levels induced by Mid1 overexpression. As Mid1 is known to facilitate translation of mRNAs harboring a CAG-repeat [20, 21], we tested whether this protein binds to *AR* mRNA in a CAG-repeat length-dependent manner. The results of an RNA pull-down assay demonstrated that Mid1 had a higher affinity for the mRNA encoding AR-97Q than AR-24Q (Fig. 2f). Furthermore, immunoprecipitation of His-tagged Mid1 followed by RT-PCR showed greater amount of *AR-97Q* mRNA bound by Mid1 as compared to *AR-24Q* mRNA (Fig. 2g). Hence, Mid1 binding to AR depends on the length of CAG-repeat expansion, leading to an increase in the protein levels of pathogenic AR potentially through translational upregulation.

### Mid1 is expressed in developing motor neurons

**Fig. 3:**
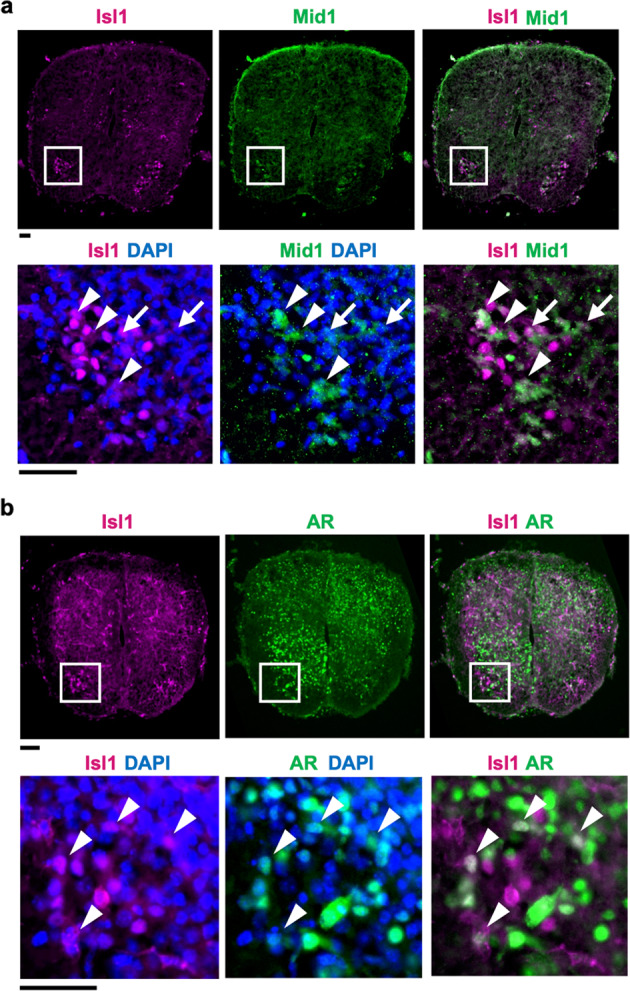
Expression of Mid1 in the developing spinal motor neurons of SBMA mice. **a** Immunostaining of Mid1 and Isl1 in the fetal mouse spinal cord. The anterior (ventral) side is toward the bottom. Boxed regions in the upper panels were magnified in the lower panels. Arrowheads and arrows indicate the overlap of strong and weak Mid1 expression with Isl1 expression, respectively. **b** Immunostaining of human AR and Isl1 in the fetal mouse spinal cord. Arrowheads indicate the overlap of nuclear AR and Isl1 expression. Scale bars 50 μm.

*Mid1* expression has been reported to be ubiquitous during early mouse development but restricted to specific organs during later development [33, 34]. We found that motor neurons showed Mid1 expression as early as the prenatal stage (Fig. 3a). Notably, the spinal cord from the prenatal AR-97Q mouse showed nuclear localization of AR (Fig. 3b), suggesting that the motor neurons were already exposed to circulating androgen during the embryonic stage. The finding of co-occurrence of Mid1 expression and AR accumulation in the nuclei of motor neurons raises the possibility that the translational upregulation of pathogenic AR by Mid1 might affect the development of motor neurons.

### Spinal cord slice cultures from the SBMA mouse model show the androgen-dependent impairment of axonogenesis

**Fig. 4:**
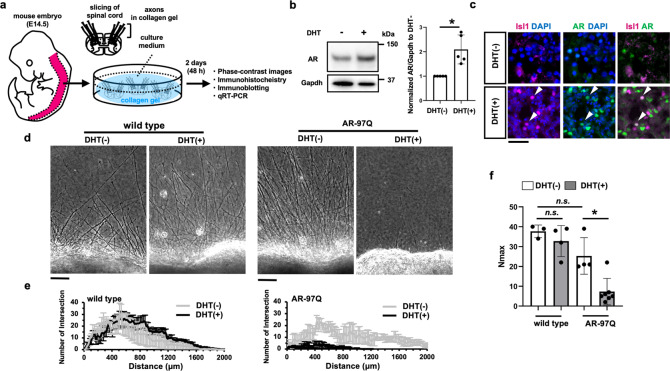
Androgen-dependent impairment of axonogenesis in the spinal cord slice culture from SBMA mice. **a** A schematic procedure for the spinal cord slice culture from AR-97Q mouse fetus at the embryonic day 14.5. Cultured spinal cord slices were collected on Day 2 and used for subsequent analyses. Phase-contrast images were acquired, focusing on the anterior side with the axons extended into the collagen gel. **b** Increased pathogenic AR protein levels. The left and right panels show the immunoblots and quantitative densitometry analyses for human AR protein levels in the spinal cord slice culture from AR-97Q mice, respectively (*n* = 5 pairs of slice cultures per group). The data were normalized to the AR protein levels without DHT treatment. Statistically, significant difference was detected by unpaired two-tailed one-sample *t*-test (*p < 0.05). **c** Co-immunostaining for nuclear human AR and Isl1, a motor neuronal marker, under DHT treatment but not without DHT in the spinal cord slice cultures from AR-97Q mice. **d** Phase-contrast images showing the effects of DHT treatment on the axonogenesis of the spinal cord slice culture from AR-97Q and wild-type male mice. **e** Quantification of the axonal distribution using Neurite-J [37]. The number of intersections was shown as a function of the distance from the center of the culture bodies. Mean ± standard error of the mean (s.e.m.) are shown (*n* = 3–7 samples per group). **f** Comparisons of the maximal number of axons detected in an image (*N*_max_) calculated from the plots in **e**. Mean ± standard deviation (s.d.) are shown. Statistically, significant difference (*p < 0.01) was detected by Tukey’s multiple comparisons test.

To examine the effects of pathogenic AR on the development of spinal motor neurons, we utilized the organotypic spinal cord slice culture obtained from AR-97Q mice, which allows the assessment of axon and motor-neuron phenotypes associated with neurodegeneration in an anatomically preserved environment [35, 36]. The spinal cord slices were embedded in the collagen gel and cultured up to 2 days (Fig. 4a). In this system, administration of DHT from the beginning of the culture resulted in greater levels of AR protein compared with the culture without DHT (Fig. 4b). Moreover, the spinal cord slices cultured with DHT showed nuclear localization of AR in the motor neurons similar to that observed in the naive embryonic spinal cord (Fig. 3b). This was also observed in the spinal cord slices cultured without DHT, albeit to a lesser extent (Fig. 4c).

We then investigated how androgen affects axonogenesis in this system. The spinal cord slices from AR-97Q mouse fetus showed substantial impairment of axonogenesis when cultured with DHT (Fig. 4d). We applied the previously reported method of image analysis to quantify axonogenesis in the spinal cord slice cultures (Fig. 4e) [37]. We found the reduction in the index for the number of axons (*N*_max_) in the cultured AR-97Q spinal cord slices treated with DHT (Fig. 4f). In the spinal cord slices from the wild-type littermates, the axonogenesis was not perturbed by the administration of DHT (Fig. 4d–f). These results showed that the androgen-dependent impairment of axonogenesis in the spinal cord slices from AR-97Q mice occurred mainly due to the toxicity of pathogenic AR.

### Axons but not cell bodies are primarily affected in the spinal cord slice culture from a mouse model of SBMA

**Fig. 5:**
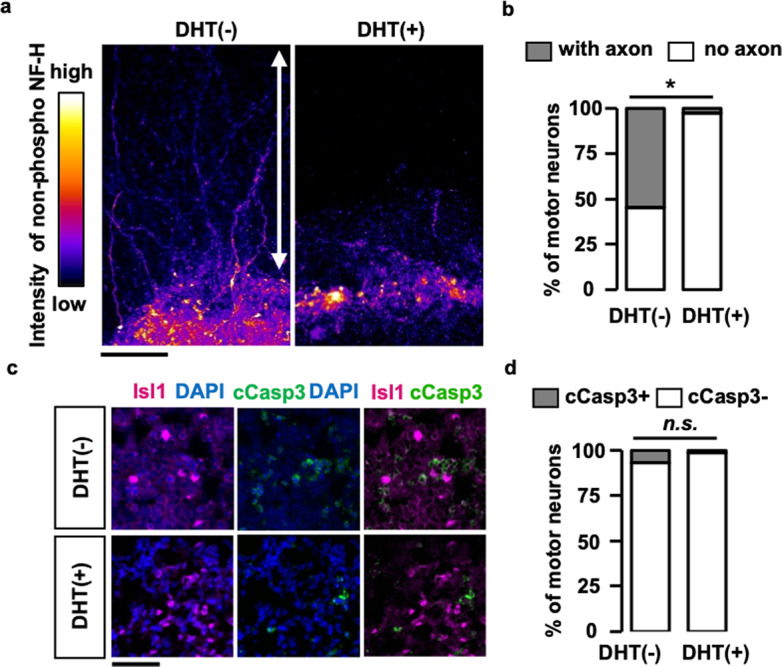
Motor axons affected in the spinal cord slice culture from SBMA mice. **a** Intensity-coded images of non-phosphorylated NF-H. The double-headed arrows indicate the region of collagen gel where axons are embedded. **b** Motor neuronal cell bodies positive for non-phosphorylated NF-H were classified either with axon or no axon. Statistically, significant difference was detected in the percentage of motor neuronal cell bodies with axons (*n* > 30 per group) by Fisher’s exact test (*p < 0.001). **c** Immunostaining of cleaved caspase 3 (cCasp3) in the spinal cord slice cultures of AR-97Q mice with or without DHT treatment. Immunostaining of cCasp3 is nearly absent in Isl1-positive motor neurons under DHT treatment. **d** Quantification of motor neuronal nuclei (*n* > 50 per group) positive for cCasp3 with or without DHT treatment. No statistically significant difference was detected by Fisher’s exact test.

To further elucidate the androgen-dependent impairment of axonogenesis in the spinal cord slice cultures from AR-97Q mice, we performed immunohistochemical staining of the spinal cord slices using an antibody against non-phosphorylated neurofilament heavy chain (NF-H), which strongly stains the cell bodies and weakly stains the axons of the motor neurons [38]. We found that the axons stained for non-phosphorylated NF-H were originated from the cell bodies stained for non-phosphorylated NF-H (Fig. 5a). Again, the genesis of the stained axons was abrogated by the administration of DHT (Fig. 5a, b). A previous study reported that caspase-3 activation is involved in cell death induced by pathogenic AR [8]. Immunostaining revealed no significant increase in the number of caspase-3-positive motor neurons (Fig. 5c, d). These results suggest that axons but not cell bodies of the motor neurons were primarily affected in the spinal cord slice culture from AR-97Q mice.

### Mid1 exacerbates the androgen-dependent impairment of axonogenesis in the spinal cord slice culture from the mouse model of SBMA

**Fig. 6:**
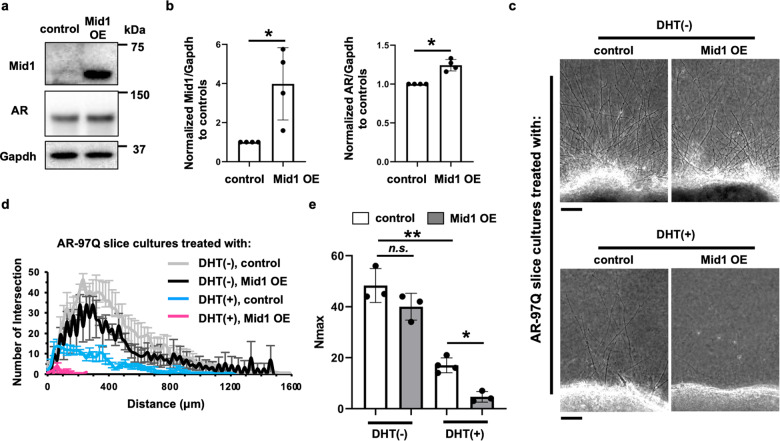
Exacerbation of axonogenesis impairment by *Mid1* overexpression in SBMA. **a** Immunoblots showing levels of Mid1, human AR, and Gapdh. **b** Quantitative densitometry analyses of Mid1 and human AR levels in **a** (*n* = 4 samples per group). **c** Phase-contrast images showing the effects of *Mid1* overexpression on the axonogenesis of the spinal cord slice culture from AR-97Q mouse fetus with or without DHT treatment. **d** For the spinal cord slice cultures from female AR-97Q female mouse fetus treated with DHT and overexpressed with GFP (control) or Mid1, plots showing the quantification of the intersection profile was obtained by Neurite-J. **e** Comparisons of the maximal number of axons detected in an image (*N*_max_). Statistically significant difference (*p < 0.05, **p < 0.001) was detected by Tukey’s multiple comparisons test.

If *Mid1* could exacerbate the androgen-dependent impairment of axonogenesis in the spinal cord slices from AR-97Q mice, *Mid1* overexpression would further impair the axonogenesis defects. To test this possibility, we introduced *Mid1* to the spinal cord slice culture by lentivirus-mediated transduction (Fig. 6a, Supplementary Fig. 3). Overexpression of *Mid1* resulted in increased pathogenic AR protein levels in a similar manner to that observed in the cellular model (Figs. 6b, 2a). Since the spinal cord slice culture from AR-97Q male mice showed a nearly complete absence of axonogenesis (Fig. 4c), we used the culture from AR-97Q female mice that showed a milder impairment of axonogenesis (Supplementary Fig. 4a, b) and thus allowed morphological analysis of axons for this purpose. *Mid1* overexpression alone without DHT treatment resulted in only moderate impairment of axonogenesis, while *Mid1* overexpression showed severe impairment of axonogenesis compared with that caused by DHT alone (Fig. 6c), which was confirmed by the quantification of axons (Fig. 6d, e). These results indicate that *Mid1* exacerbates the androgen-dependent impairment of axonogenesis in the spinal cord slice culture from AR-97Q mice, especially under androgen exposure.

### Mid1 knockdown ameliorates the androgen-dependent impairment of axonogenesis in the spinal cord slice culture from a mouse model of SBMA

**Fig. 7:**
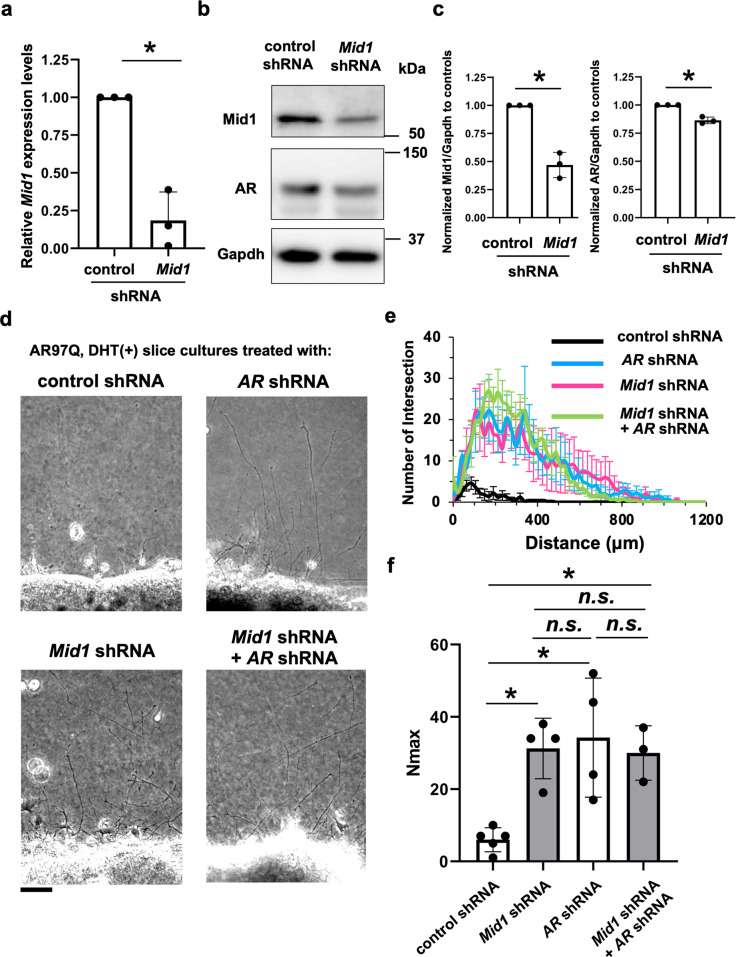
Amelioration of axonogenesis impairment by *Mid1* knockdown in SBMA. **a** Quantification of *Mid1* expression levels in the spinal cord slice cultures from wild-type mouse fetus transfected with lentivirus expressing control shRNA or *Mid1* shRNA. Statistically, significant difference (*p < 0.05) was detected by an unpaired two-tailed one-sample *t*-test. **b** Immunoblots showing levels of Mid1, AR, and Gapdh in the spinal cord slice culture from AR-97Q male mouse fetus transfected with control shRNA or *Mid1* shRNA. **c** Quantitative densitometry analyses of Mid1 and human AR levels in **a** (*n* = 3 samples per group). **d** Phase-contrast images showing the effects of *Mid1 and/or human AR* knockdowns on the axonogenesis of the spinal cord slice culture from AR-97Q mouse fetus treated with DHT. **e** For the spinal cord slice cultures from AR-97Q male mouse fetus treated with DHT and shRNA (control, *Mid1*, human *AR*, or both *Mid1* and *human AR*), plots showing the quantification of the intersection profile was obtained by Neurite-J. **f** Comparisons of the maximal number of axons detected in an image (*N*_max_). Statistically, significant differences (*p < 0.05) were detected by Tukey’s multiple comparisons test.

We further examined whether *Mid1* knockdown rescues the axonogenesis defects. We first confirmed that knockdown of *Mid1* did not affect axonogenesis in the spinal cord slice culture from wild-type mice (Supplementary Fig. 5). Next, in the spinal cord slice culture from AR-97Q male mice that had severe impairment of axonogenesis than female mice (Fig. 4d, Supplementary Fig. 4), we found that *Mid1* knockdown decreased the pathogenic AR protein levels (Fig. 7a–c). Furthermore, *Mid1* knockdown increased the protein levels of PP2Ac, a ubiquitination target of Mid1 involved in the translational regulatory complex of mTOR [39] (Supplementary Fig. 6). As expected, *Mid1* knockdown ameliorated the axonogenesis defects (Fig. 7d–f), suggesting that endogenous *Mid1* is responsible for the androgen-dependent impairment of axonogenesis in the spinal cord slice culture from AR-97Q mice. In addition, knockdown of the human *AR* transgene rescued the impairment of axonogenesis regardless of co-silencing of *Mid1* (Fig. 7d–f, Supplementary Figs. 7, 8). Overall, Mid1 expressed in motor neurons played a crucial role in the androgen-dependent impairment of axonogenesis in the spinal cord slice culture from AR-97Q mice.

## Discussion

The present study demonstrates that Mid1, a microtubule-associated RNA binding protein exclusively expressed in the motor neurons of the spinal cord, interacts with CAG-repeat expanded *AR* mRNA and increases the protein levels of pathogenic AR in both cellular and organotypic slice culture models of SBMA. The transcriptional upregulation of *Mid1* by pathogenic AR induced the translational upregulation of pathogenic AR in the spinal cord of early symptomatic SBMA mice and accelerated the motor neuron pathology in SBMA. Interestingly, AR has been shown to repress the transcription of *Mid1*, forming a negative feedback loop leading to suppression of AR protein levels [22]. Hence, a loss-of-function property of pathogenic AR might affect this negative feedback regulation, resulting in increased *Mid1* transcription. The bi-directional feedbacks between pathogenic AR and Mid1 could contribute to the vulnerability of motor neurons in SBMA.

Although SBMA is an adult-onset disease, studies in the primary culture of embryonic motor neurons from the mouse model of SBMA have suggested that both transcriptional and translational dysregulation by pathogenic AR impair motor neuron homeostasis as an early dysregulation during development [17, 40]. Our finding that pathogenic AR accumulated in the nuclei of embryonic motor neurons in AR-97Q mouse fetus, probably due to prenatal exposure to androgen [41], further suggests that pathogenic AR begins affecting motor neurons during embryonic stage itself. Substantial impairment of axonogenesis in the spinal cord slice cultures from AR-97Q mice in the presence of androgen may be attributed to, at least in part, the degree of pathogenic AR accumulation during embryogenesis. Regarding slice cultures, the greater impairment of axonogenesis in males compared to the females indicates the role of prenatal exposure to androgens in SBMA pathogenesis, especially in males. Moreover, the spinal cord slices from AR-97Q mice cultured without androgen showed axonogenesis levels similar to wildtype (Fig. 4f), suggesting that the effect of embryonically accumulated pathogenic AR could be offset by androgen deprivation. In our previous study [9], this reversibility was consistent with the rescue of axonal dysfunctions in AR-97Q mice by androgen depletion. Considering the role of Mid1 in the translation of pathogenic AR, targeting Mid1 may be effective as therapy against motor axonal vulnerability in SBMA even at an adult stage.

Our study indicates that the impairment of axonal growth by pathogenic AR partly depends on the translational upregulation of pathogenic AR by Mid1. It has also been reported that axonal growth could be directly regulated by Mid1-PP2A system [24, 42]. Still, this mechanism is unlikely to explain the axonogenesis defects in the spinal cord slice cultures from AR-97Q mice for three reasons. First, the impairment of axonogenesis in the spinal cord slice culture from AR-97Q mice is dependent on androgen levels but not on Mid1 alone (Figs. 4, 6). Second, axonogenesis in spinal cord slices from wild-type mice was not affected by the knockdown of *Mid1* (Supplementary Fig. 3). Third, double knockdown experiments of *Mid1* and *AR* suggest that the impairment of axonogenesis by Mid1 depends on the pathogenic AR (Fig. 7). Another interesting feature of Mid1 is its association with microtubules. Mid1 itself is transported along the axonal microtubule in a manner dependent on the dephosphorylation by PP2A [43]. Thus, the association of Mid1 with microtubule might also contribute to the effect of pathogenic AR on axonal transport via the increase in its local translation of pathogenic AR. In light of previous reports, cytoplasmic oligomers of pathogenic AR disrupt axonal transport, thereby impairing the neurite growth [8, 9]. Accordingly, increased translation of pathogenic AR by Mid1 in the vicinity of the microtubule may disrupt axonal transport and be involved in the substantial impairment of axonogenesis observed in the present study. If this mechanism involves the axonal vulnerability of motor neurons observed in the in vivo settings [6, 7, 9] needs to be explored in future studies.

Our study demonstrated that the embryonic spinal cord slice culture is a useful model for elucidating the axonal vulnerability in SBMA, which might reflect the early dysregulation of motor neurons in vivo. In the organotypic slice culture, axonogenesis is supported by the surrounding extracellular matrix and glial cells [35], in addition to rich trophic support by the culture medium. These conditions allowed axonogenesis of wild-type or androgen-untreated SBMA motor neurons but could not support axonogenesis of androgen-treated SBMA motor neurons that may have experienced higher cytotoxic stresses. In this sense, the analysis of axonogenesis in the spinal cord slice culture may serve as stress load testing for SBMA motor neurons. Also, spinal cord slice cultures were easily transduced with a lentivirus vector without the requirement of its in vivo administration, especially for embryos. This advantageous property further enabled us to elucidate the mechanism of androgen-dependent neuronal and axonal vulnerabilities of SBMA motor neurons. Studies of the developmental origin of functional deficits in motor axons using spinal cord slice culture models will advance our understanding of neuronal pathogenesis and lead to the development of targeted therapeutics in SBMA.

### Ethics

All animal experiments were performed in accordance with the National Institute of Health Guide for the Care and Use of Laboratory Animals and with the approval of the Nagoya University Animal Experiment Committee.

## Supplementary information

Reproducibility checklist

Original Data File

Supplemental Data

## Data Availability

The datasets generated during and/or analyzed during the current study are available from the corresponding author on reasonable request.
