# Supplementary figures and images for: Mid1 is associated with androgen-dependent axonal vulnerability of motor neurons in spinal and bulbar muscular atrophy
Source: Cell Death Dis. 2022 Jul 13;13(7):601. doi: 10.1038/s41419-022-05001-6 (PMC9276699; doi:10.1038/s41419-022-05001-6)

Fig.2

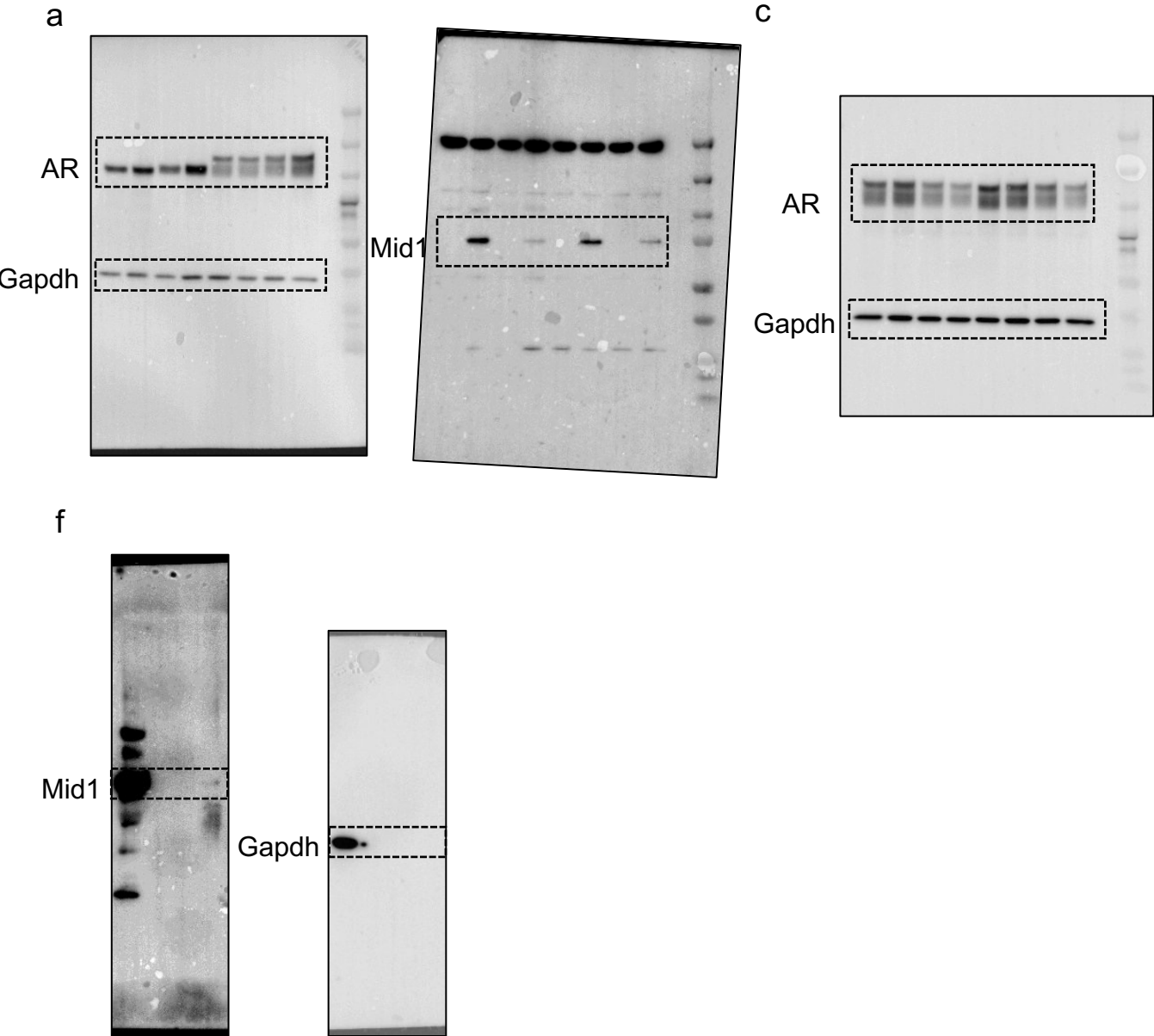

Fig.4

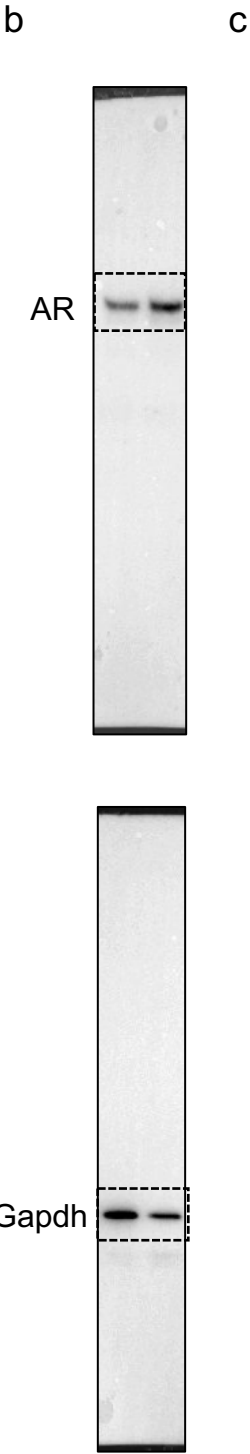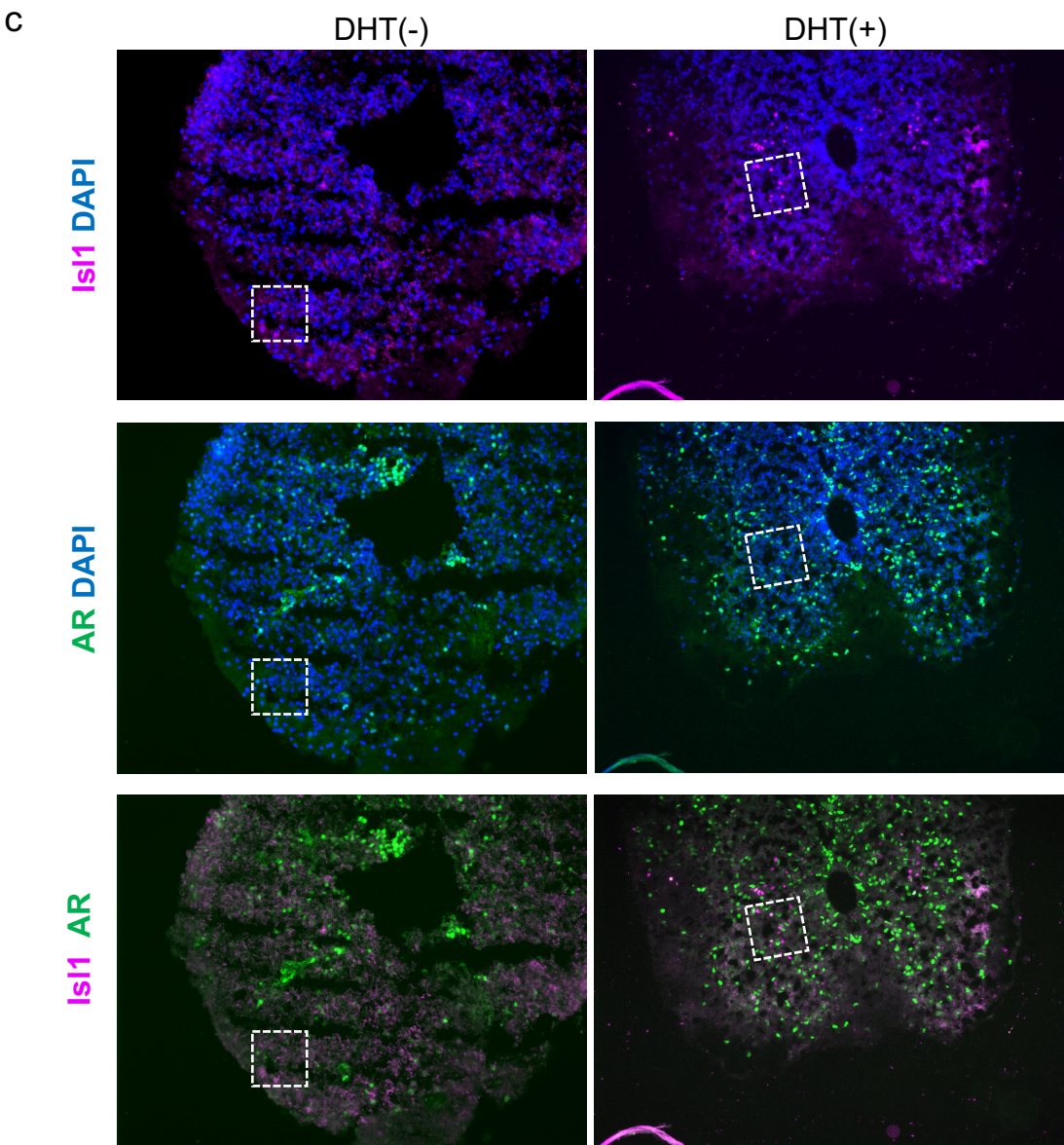

Fig.4

d

DHT(-)

DHT(+)

wildtype

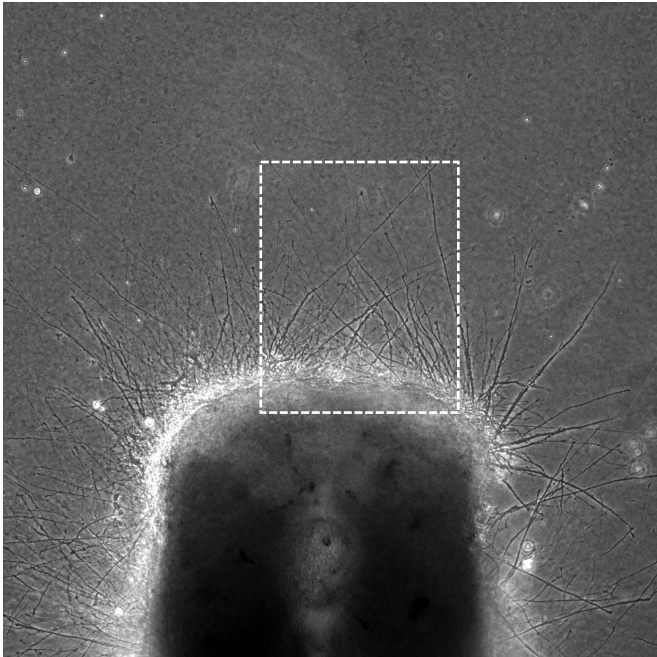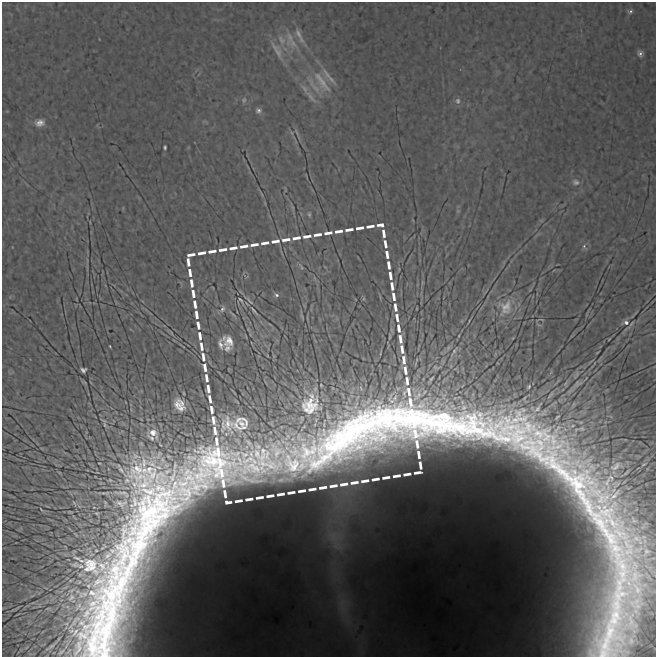

AR-97Q

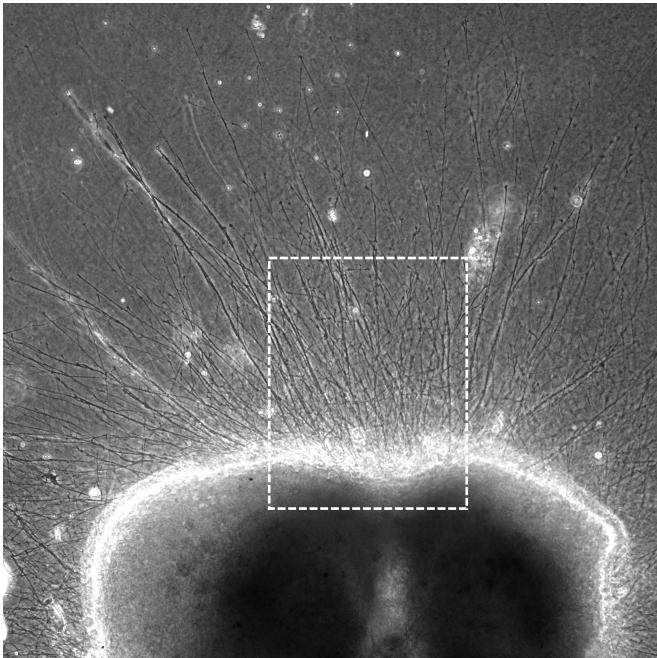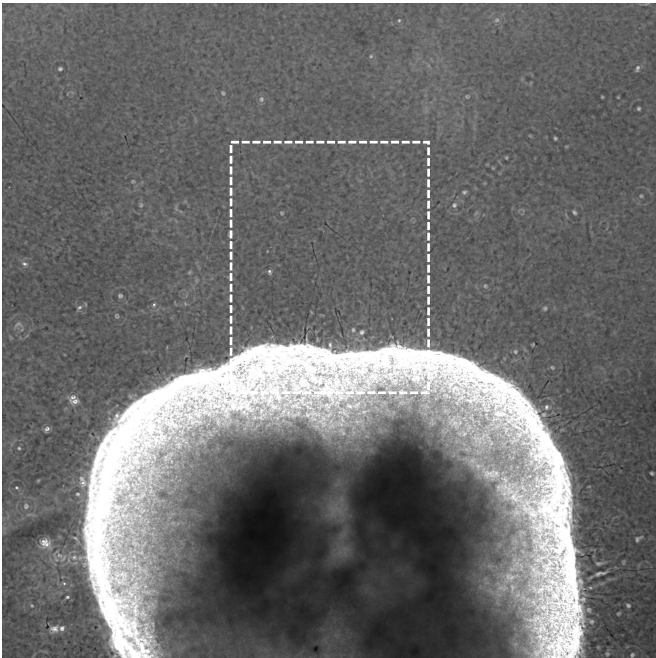

Fig.5

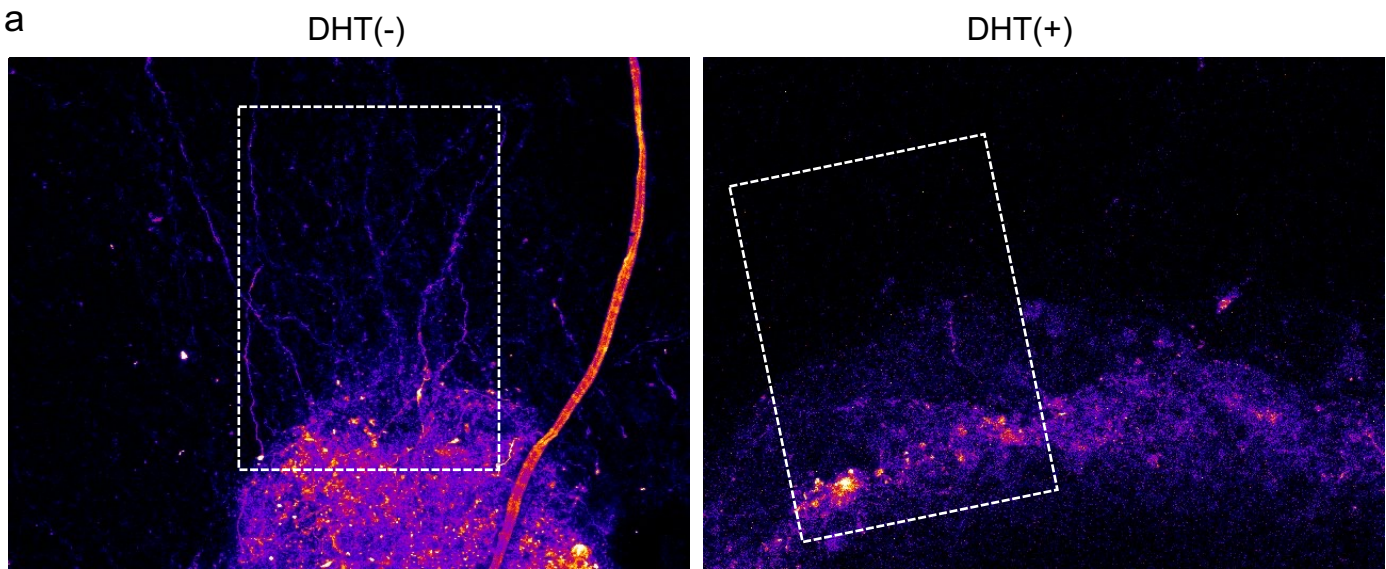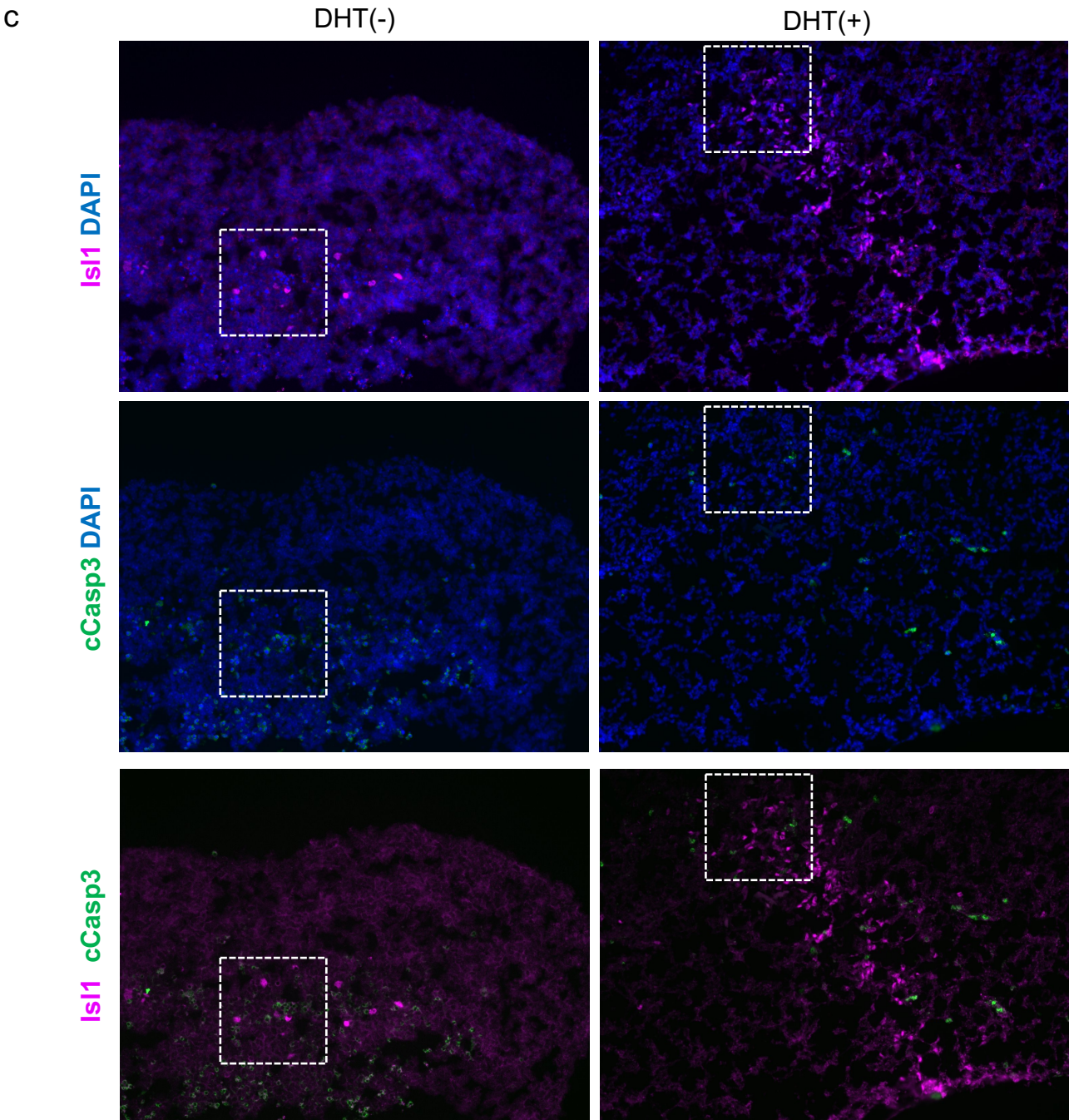

Fig.6

a

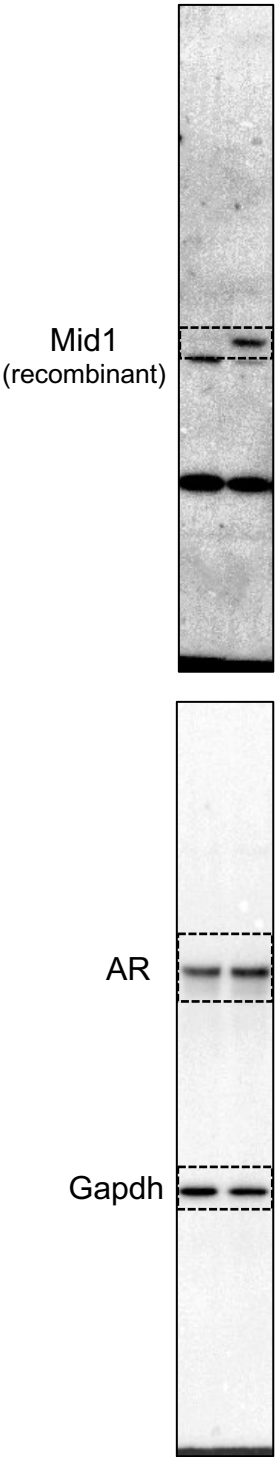

c

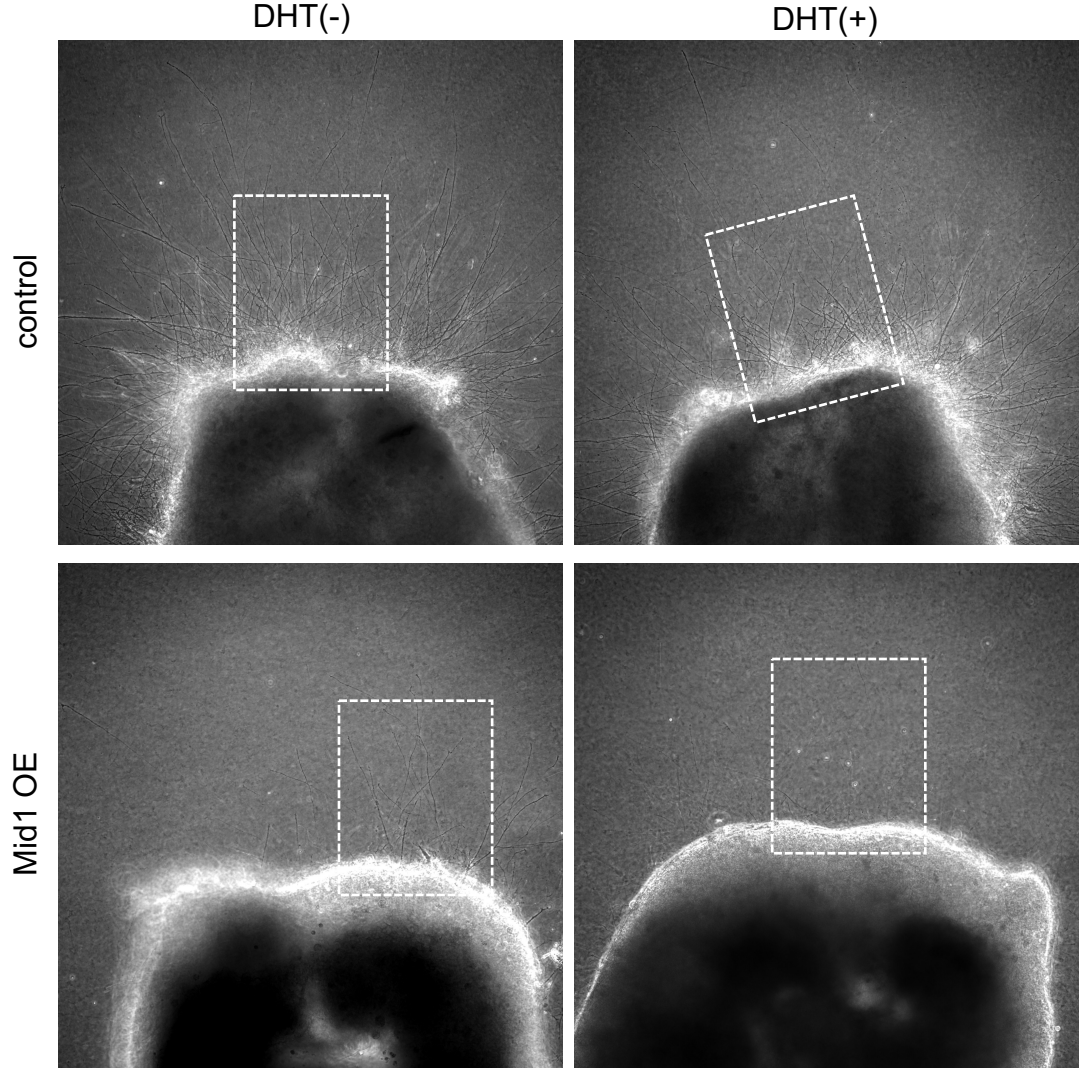

Fig.7

a

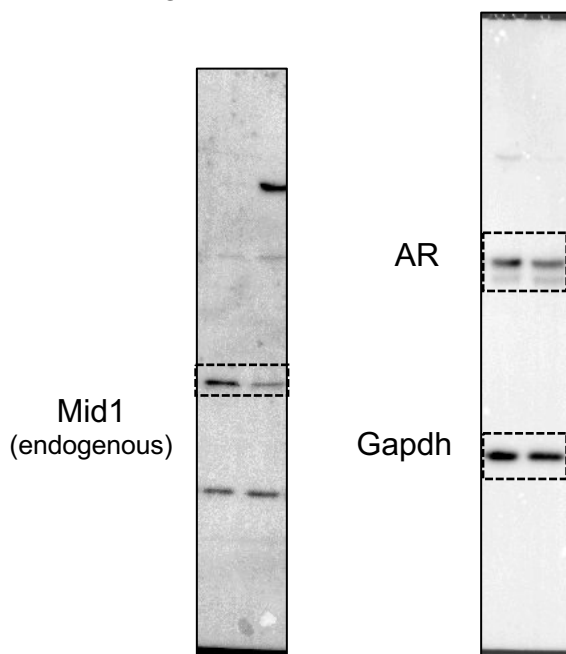

d

control

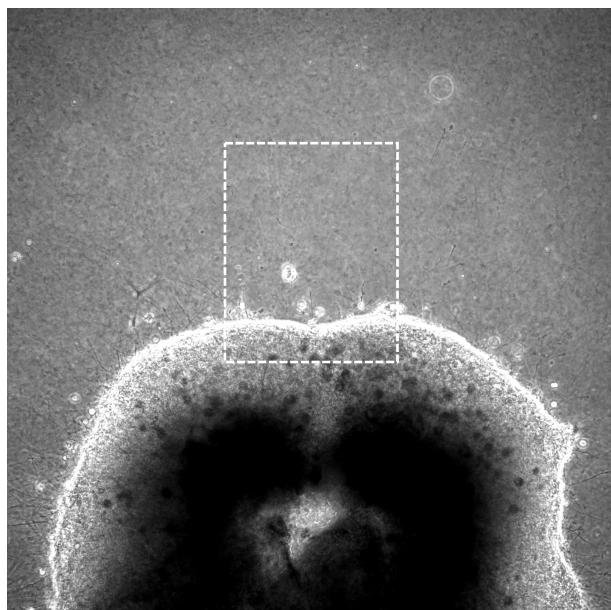

AR shRNA

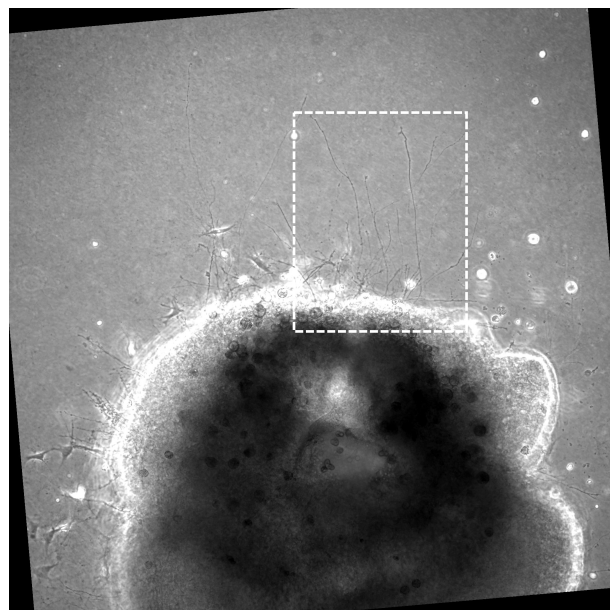

Mid1 shRNA

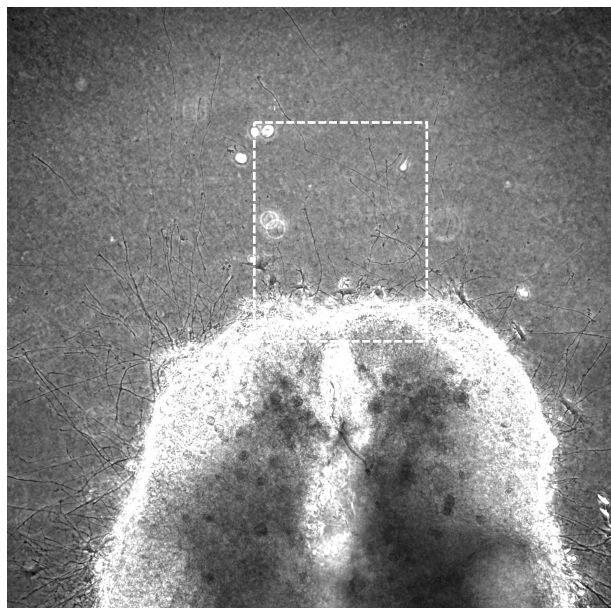

Mid1 shRNA + AR shRNA

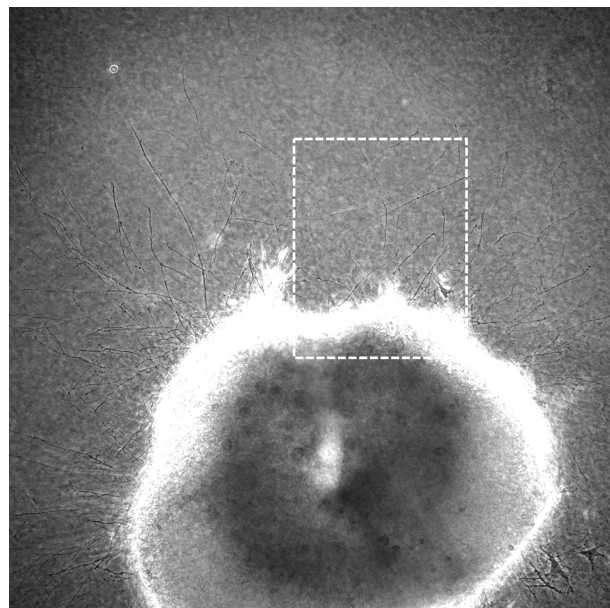

Supplement: Supplementary file 2 — Original Data File [file 41419_2022_5001_MOESM2_ESM.pdf]
