# Supplementary material for: Mid1 is associated with androgen-dependent axonal vulnerability of motor neurons in spinal and bulbar muscular atrophy
Source: Cell Death Dis. 2022 Jul 13;13(7):601. doi: 10.1038/s41419-022-05001-6 (PMC9276699; doi:10.1038/s41419-022-05001-6)
Supplement: Supplementary file 3 — Supplemental Data [file 41419_2022_5001_MOESM3_ESM.docx]

**Supplemental Data**

**Mid1 is associated with androgen-dependent axonal vulnerability of motor neurons in spinal and bulbar muscular atrophy**

Yosuke Ogura, Kentaro Sahashi, Tomoki Hirunagi, Madoka Iida, Takaki Miyata and Masahisa Katsuno

**Contents:**

Eight Supplementary Figures (Supplementary Figure 1, Supplementary Figure 2, Supplementary Figure 3, Supplementary Figure 4, Supplementary Figure 5, Supplementary Figure 6, Supplementary Figure 7, Supplementary Figure 8)

One Supplementary Table (Supplementary Table 1)

**Supplementary materials and methods
Immunohistochemistry of autopsy samples**Autopsy specimens of the lumbar spinal cord were obtained from patients with genetically confirmed SBMA and control subjects. The subjects with cerebellar hemorrhage and suspected Guillain-Barre syndrome were used as controls for the spinal cord samples. The collection and use of human tissues in this study were approved by the Ethics Committee of Nagoya University Graduate School of Medicine. The specimens were dissected, post-fixed with 10% phosphate-buffered formalin and processed for paraffin embedding. Sections with 6 μm thickness were prepared from paraffin-embedded tissues using cryo-sectioned and were stained using anti-MID1 pAb (Abcam, 1:5000) and a secondary antibody labeled with a polymer as part of the Envision + system containing horseradish peroxidase (Dako Cytomation, Gostrup, Denmark). The sections were co-stained with hematoxylin and eosin for counter staining. Images of immunohistochemically stained sections were photographed using an optical microscope (BX51, Olympus, Tokyo, Japan).
 **
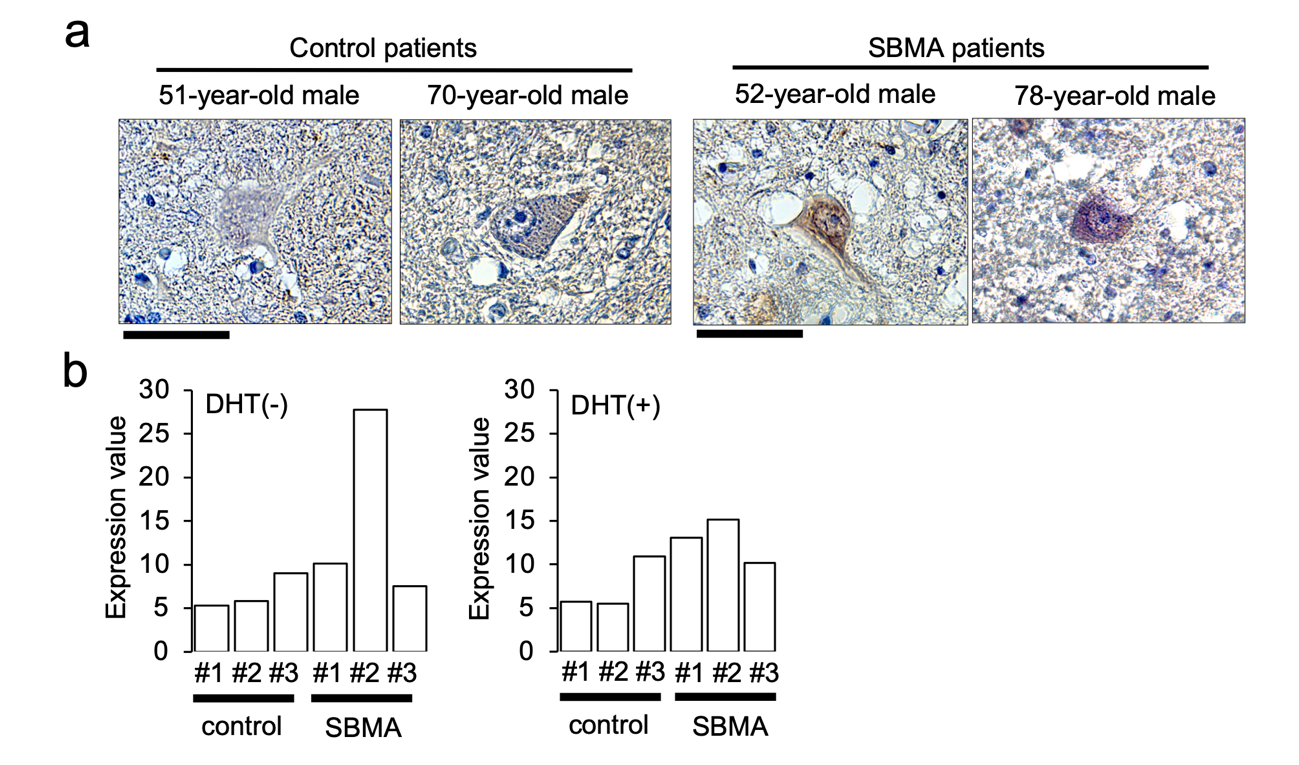

Supplementary Figure 1 Expression of Mid1 in SBMA patients and induced pluripotent stem cell (iPSC)-derived motor neurons from patients with SBMA.**

**a** Immunostaining of Mid1 in spinal cord samples from control subjects and patients with SBMA. Scale bars 50 μm. **b** Expression values (TPM) are shown for publicly available RNA-seq data (GSE138053) obtained from iPSCs-derived motor neurons from control and patients with SBMA cultured with or without DHT^31^.

**
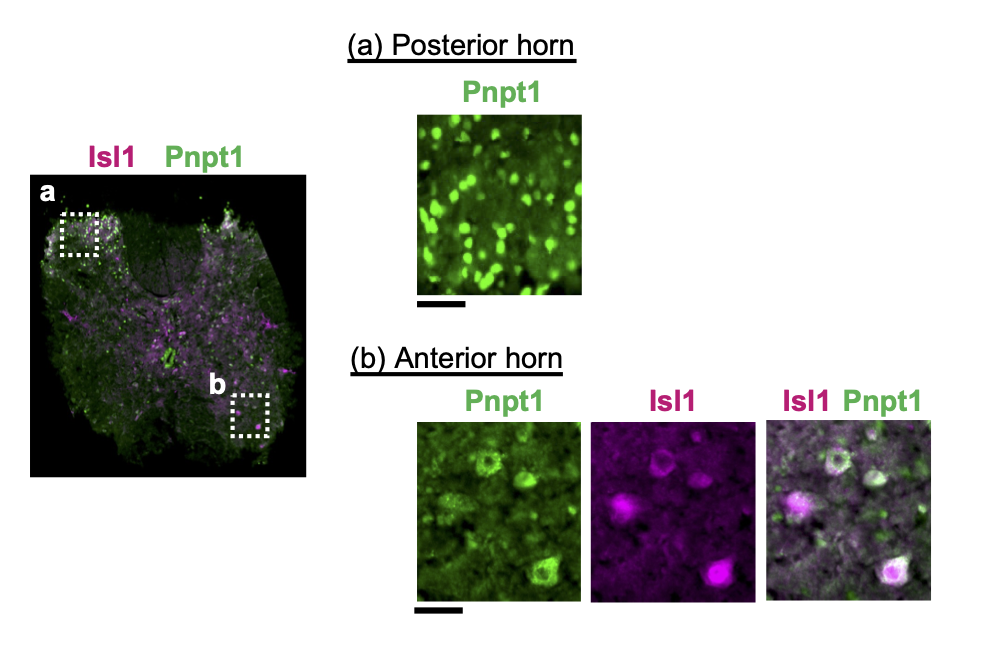
**

**Supplementary Figure 2 Expression of Pnpt1 in mouse spinal cord.**Immunostaining of Pnpt1 the of 10 weeks spinal cord of wild type mouse. Isl1 is a motor neuron marker. Pnpt1 expression was detected in motor neurons (b) as well as the other cell types in the posterior horn (a). Scale bars 50 μm.

**
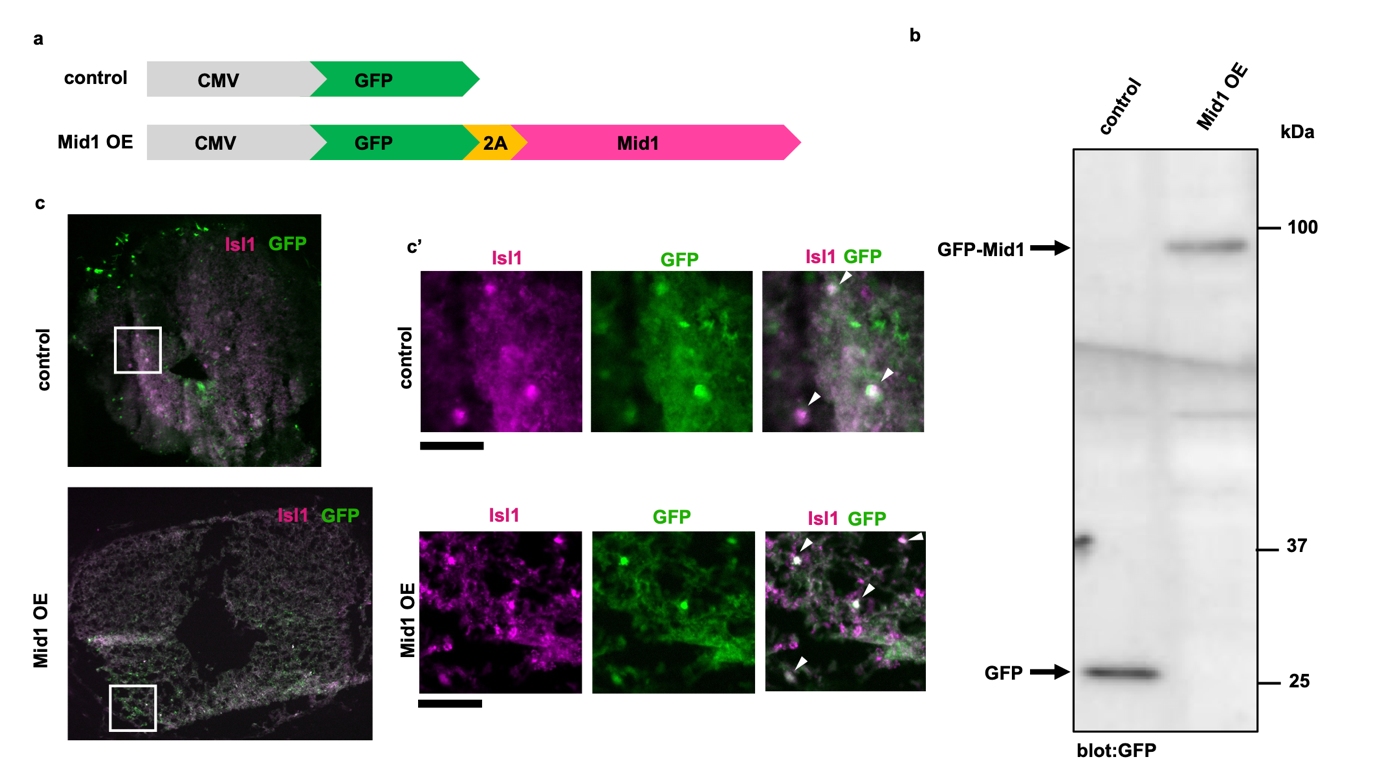
**

**Supplementary Figure 3 Lentivirus-mediated transduction of GFP and Mid1 in the spinal cord slice culture.**

**a** Schematic diagram of the constructs used to express GFP and Mid1 under the control of cytomegalovirus (CMV) promoter. GFP and Mid1 was linked by the 2A peptide. **b** Immunoblots with GFP antibody showing levels of GFP and GFP-Mid1 fusion in the cultured spinal cord slices transduced with lentivirus expressing GFP (control) or GFP-Mid1 (Mid1 OE). **c** Immunostaining of Isl1 and GFP in the cultured spinal cord slices transduced with lentivirus expressing GFP (control) or GFP-Mid1 (Mid1 OE). Arrowheads indicate the overlap of Isl1 and GFP expression. The anterior (ventral) side is toward the bottom. Boxed regions in **c** were magnified in the panels in **c’**. Scale bars 50 μm.

**
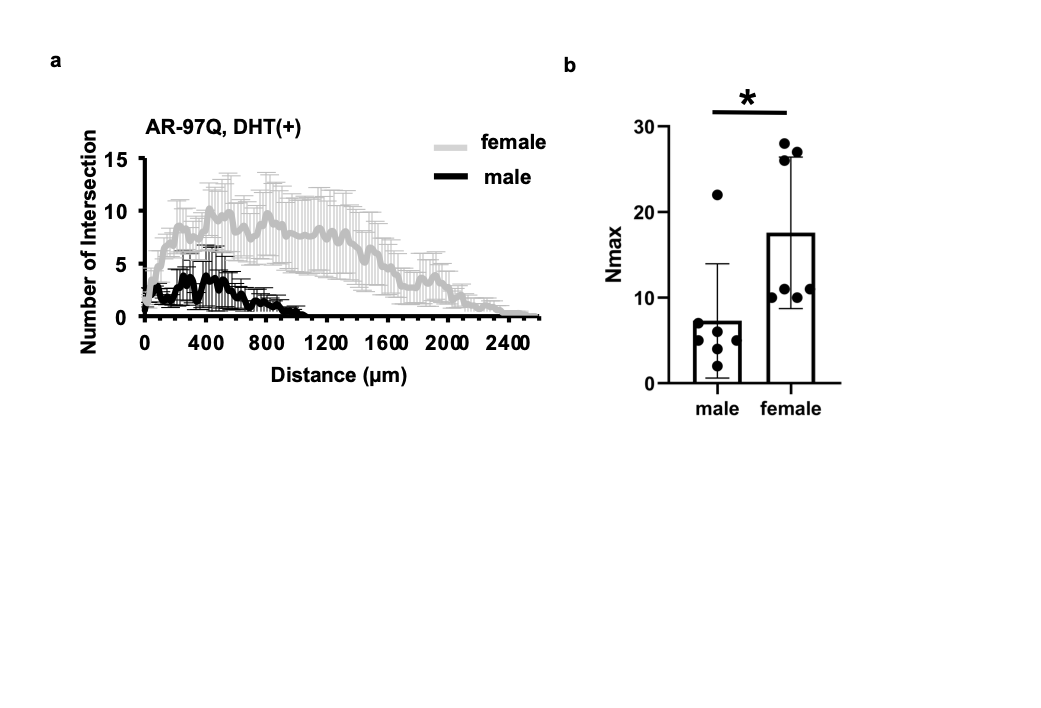
**

**Supplementary Figure 4 Sexual differences in axonogenesis impairment in SBMA.**

**a** Images of spinal cord slice cultures from AR-97Q male and female mouse fetus treated with DHT were used for the quantification of the intersection profile by Neurite-J (*n* = 7 samples per group). **b** Comparisons of the maximal number of axons detected in an image (N_max_). Significant difference (**p* < 0.05) was detected by unpaired two-tailed two-sample *t*-test.

**
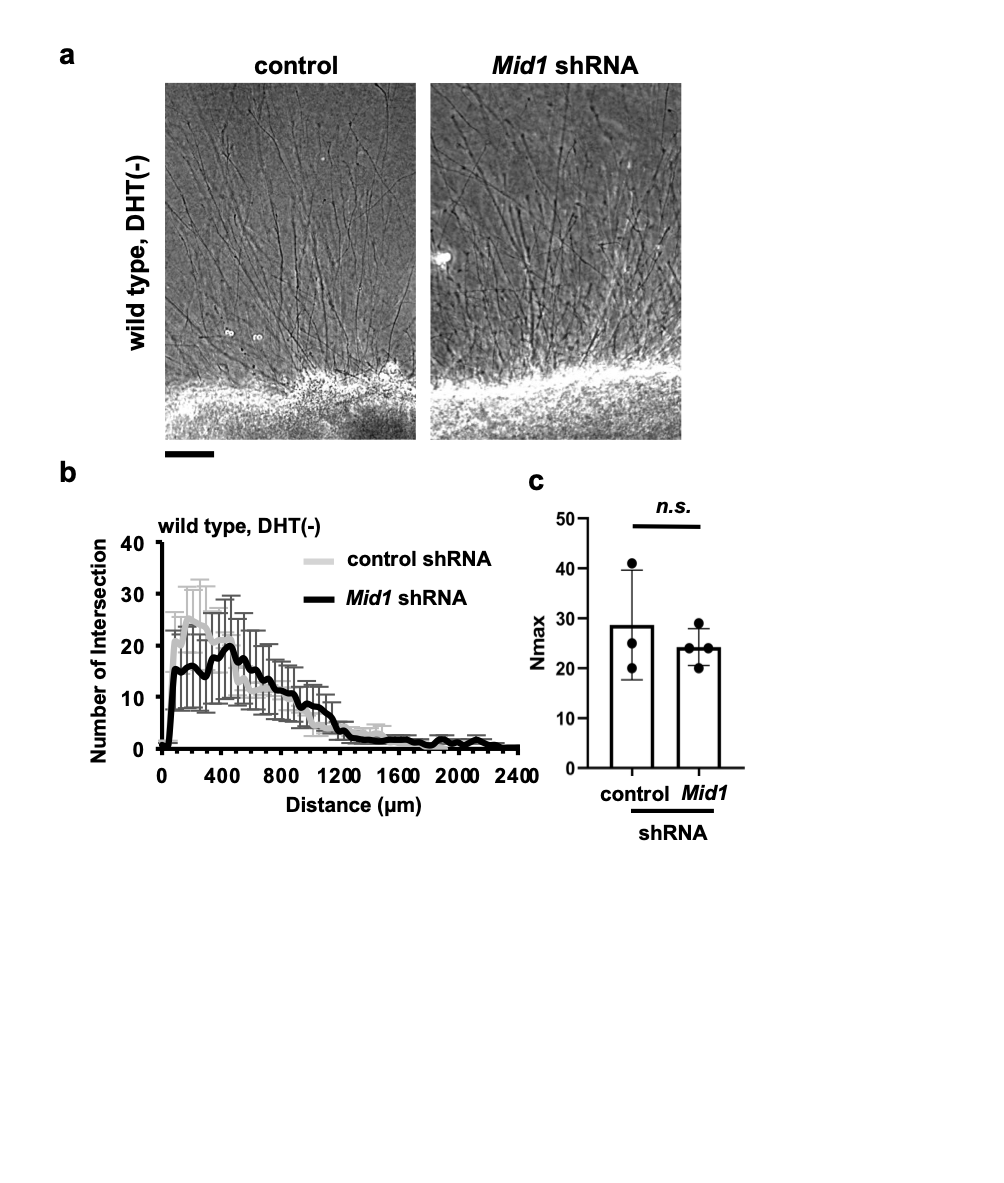
**

**Supplementary Figure 5 Effect of *Mid1* knockdown on axonogenesis in wild-type.
a** Phase-contrast images showing the effects of *Mid1* knockdown on the axonogenesis of the spinal cord slice culture from the wild-type mouse fetus cultured without DHT. **b** Images of spinal cord slice cultures from wild-type male mouse fetus were used for the quantification of the intersection profile by Neurite-J (*n* = 3 and 4 samples for control and *Mid1* knockdown, respectively). **c** Comparisons of the maximal number of axons detected in an image (N_max_). No statistically significant difference was detected by unpaired two-tailed two-sample *t*-test.


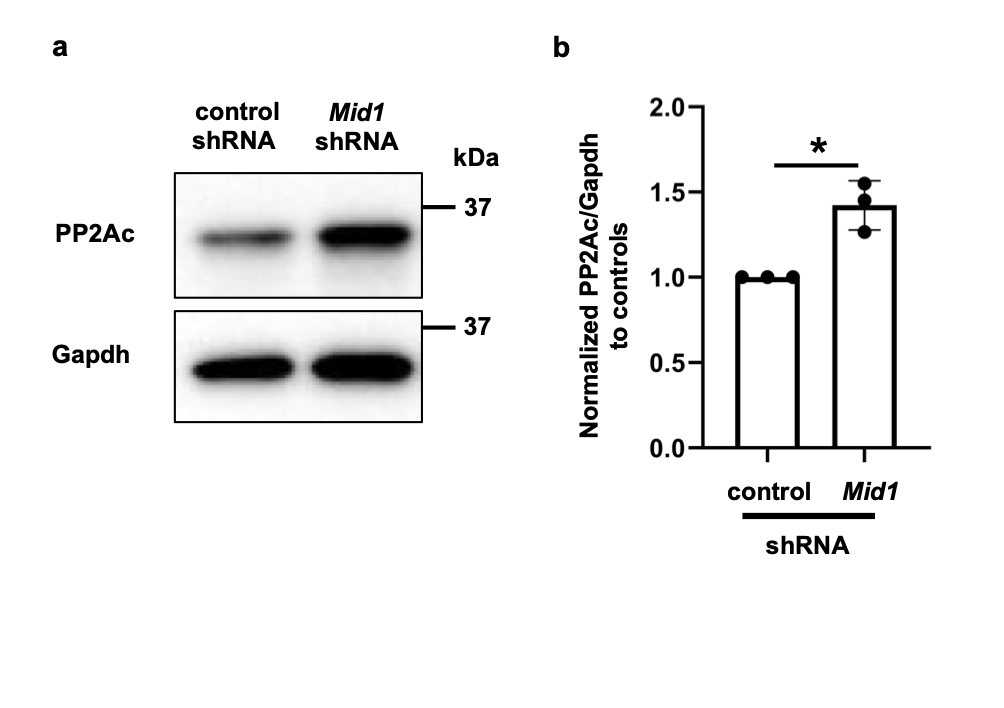


**Supplementary Figure 6 Effect of *Mid1* knockdown on PP2Ac protein levels.**

**a** Immunoblots showing levels of PP2Ac and Gapdh in the cultured spinal cord slices from AR-97Q mouse fetus transduced with lentivirus expressing control shRNA or *Mid1* shRNA. **b** Quantitative densitometry analyses of PP2Ac protein levels in **a** (*n* = 3 samples per group). The data were normalized to the PP2Ac level in the controls.

**
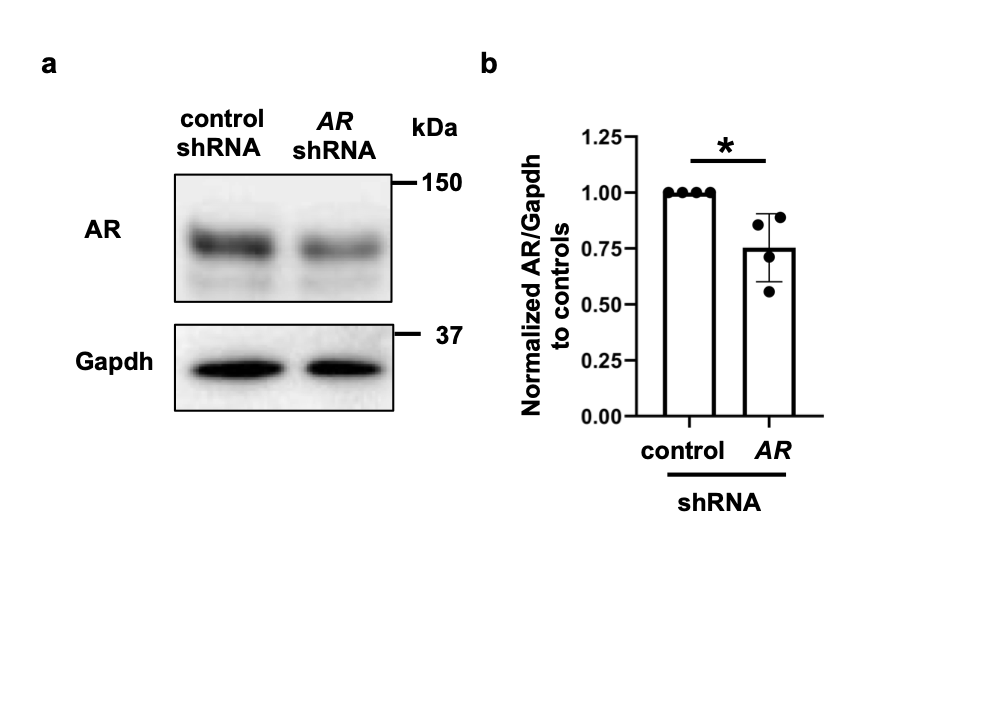
 Supplementary Figure 7 Knockdown of human *AR* transgene.**

**a** Immunoblots showing levels of human AR and Gapdh in the cultured spinal cord slices from AR-97Q male mouse fetus transduced with lentivirus expressing control shRNA or human *AR* shRNA. **b** Quantitative densitometry analyses of human AR protein levels in **a** (*n* = 4 samples per group). The data were normalized to the AR level in the controls.

**
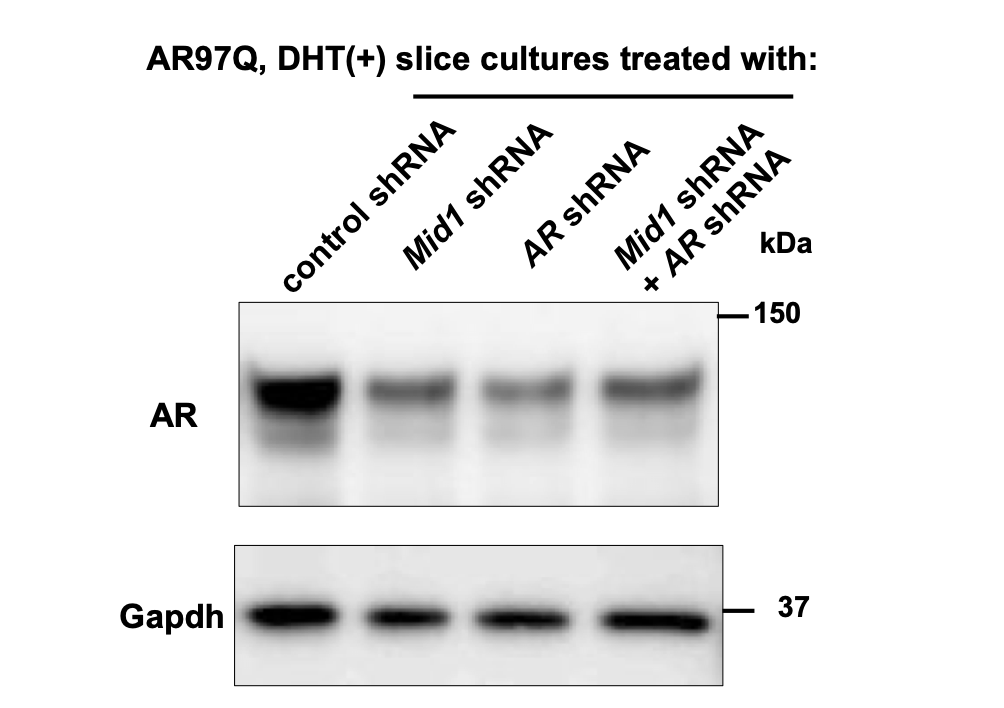
Supplementary Figure 8 Effect of simultaneous knockdowns of *Mid1* and human *AR*.**

Immunoblots showing levels of human AR and Gapdh in the cultured spinal cord slices from AR-97Q male mouse fetus treated with DHT and transduced with the indicated lentiviruses.

|  | **Gene symbol** | **GeneCards description** | **logFC** | **FDR** | **Stage** |
| --- | --- | --- | --- | --- | --- |
| **Upregulated** | ***Mid1*** | **RING-type E3 ubiquitin transferase Midline-1** | **2.49** | **0.050** | **10-12 weeks (early stage)** |
|  | ***Pnpt1*** | **Polyribonucleotide nucleotidyltransferase 1** | **2.47** | **0.005** | **7-9 weeks (before-onset stage)** |
|  | ***Map1b*** | **Microtubule associated protein 1B** | **1.45** | **0.001** | **7-9 weeks (before-onset stage)** |
|  | ***Ap2b*** | **Adaptor related protein complex 2 subunit β** | **1.04** | **0.050** | **10-12 weeks (early stage)** |
|  |  |  |  |  |  |
| **Downregulated** | ***Zfp704*** | **Zinc finger protein 704** | **-1.05** | **0.013** | **7-9 weeks (before-onset stage)** |
|  | ***Fggy*** | **FGGY carbohydrate kinase domain containing** | **-1.33** | **0.029** | **7-9 weeks (before-onset stage)** |
|  | ***Anks1b*** | **Ankyrin repeat and sterile alpha motif domain containing 1B** | **-1.33** | **0.044** | **7-9 weeks (before-onset stage)** |

**Supplementary Table 1 Dysregulated genes in the spinal cord of SBMA mice.**

A list of genes whose expression was up-regulated or down-regulated more than two-fold in the spinal cord of AR-97Q mice compared to that of AR-24Q mice^4^. Motor neuronal expression was examined using the single-cell transcriptional atlas^27^. See details for the main text.
